# Arthroscopic subacromial decompression versus placebo surgery for subacromial pain syndrome: 10 year follow-up of the FIMPACT randomised, placebo surgery controlled trial

**DOI:** 10.1136/bmj-2025-086201

**Published:** 2025-12-02

**Authors:** Kari Kanto, Mathias Bäck, Thomas Ibounig, Robert Björkenheim, Antti Malmivaara, Tomasz Czuba, Jari Inkinen, Juha Kalske, Vesa Savolainen, Ilkka Sinisaari, Pirjo Toivonen, Simo Taimela, Teppo L N Järvinen, Mika Paavola, Finnish Shoulder Impingement Arthroscopy Controlled Trial (FIMPACT) Investigators, Jonas Ranstam, Jarkko Pajarinen, Sikri Tukiainen, Kalevi Hietaniemi, Vesa Lepola, Jyrki Salmenkivi, Mikko Salmela, Timo Järvelä, Janne Lehtinen, Ville Haapamäki, Heikki Kolehmainen, Mikael Salmela, Tarja Kunnala, Sami Niskanen, Hanna-Mari Laiho, Leena Kangas-Viri, Leena Caravitis, Sanna Hokkanen, Marketta Rautanen, Sari Karesvuori, Soile Lindholm, Eero Hölli, Lena Laine, Esa Läärä

**Affiliations:** 1 Finnish Centre for Evidence-Based Orthopaedics (FICEBO), University of Helsinki, Helsinki, Finland; 2 Department of Orthopaedics and Traumatology, Tampere University Hospital, Tampere, Finland; 3 Department of Orthopaedics and Traumatology, Helsinki University Hospital, Haartmaninkatu 4, PO Box 320, 00029 HUS, Helsinki, Finland; 4 National Institute for Health and Welfare, Centre for Health and Social Economics, Helsinki, Finland; 5 Orton Orthopaedic hospital, Helsinki, Finland; 6 Department of Molecular and Clinical Medicine, University of Gothenburg, Gothenburg, Sweden; 7 Physiotherapy centre Fysios Mehiläinen, Tampere, Finland; 8 Department of Orthopaedics and Traumatology, Helsinki University Hospital, Jorvi hospital, Espoo, Finland; 9 Pihlajalinna Hospital, Helsinki, Finland; 10 Terveystalo Hospital, Helsinki, Finland

## Abstract

**Objective:**

To assess the long term efficacy of arthroscopic subacromial decompression (ASD) versus placebo surgery (diagnostic arthroscopy) and exercise therapy in patients with subacromial pain syndrome.

**Design:**

Randomised, placebo surgery controlled trial.

**Setting:**

Orthopaedic department of three public hospitals in Finland.

**Participants:**

210 adults aged 35 to 65 years with symptoms consistent with subacromial pain syndrome for more than three months, enrolled from 1 February 2005 with 10 year follow-up to 20 September 2023. Participants and outcome assessors were blinded to group allocation in the primary (ASD versus placebo surgery) comparison.

**Interventions:**

ASD, placebo surgery, and exercise therapy (1:1:1). Exercise therapy was used as a pragmatic comparator.

**Main outcome measures:**

The primary outcomes were shoulder pain at rest and on arm activity, both assessed at 10 years using a visual analogue scale (VAS, ranging from 0 to 100, with 0 denoting no pain). The minimally important difference was defined as 15. A mixed model repeated measures analysis of variance was used, treating participants as random factors, incorporating baseline values as covariates.

**Results:**

Participants were randomly assigned to ASD (n=59), placebo surgery (n=63), and exercise therapy (n=71). Of these, a total of 168 participants (87%) completed the 10 year follow-up. In the primary intention-to-treat analysis (ASD versus placebo surgery), no between group differences were observed for the two primary outcomes at 10 years: the mean difference between groups (ASD minus placebo surgery) was −1.5 points (95% confidence interval (CI) −8.6 to 5.6) in VAS pain score at rest and −3.2 points (−13.0 to 6.5) in VAS pain score on arm activity. No significant between group differences were found for any of the secondary outcomes or adverse events. In the pragmatic comparison, the mean difference between groups (ASD minus exercise therapy) was −4.0 points (−11.0 to 3.0) in VAS pain score at rest and −9.4 points (−19.0 to 0.3) in VAS pain score on arm activity. No significant between group differences were observed for the secondary outcomes or adverse events.

**Conclusion:**

In patients with subacromial pain syndrome, ASD offered no benefit over placebo surgery or exercise therapy during 10 year follow-up.

**Trial registration:**

ClinicalTrials.gov NCT00428870.

**Figure:**
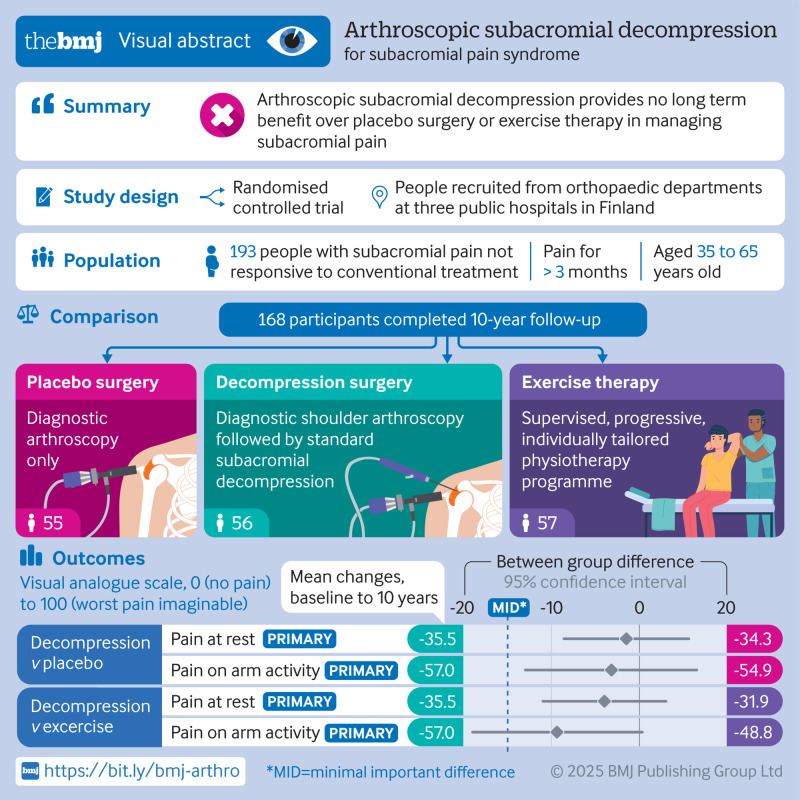


## Introduction

In England, nearly 30 000 arthroscopic subacromial decompression (ASD) surgeries are performed annually for shoulder pain, with rates even higher in other countries,^1^ ^2^ ^3^ making ASD one of the most common orthopaedic procedures.^4^ Since 2007-08, the cumulative cost of ASD surgeries to the NHS has surpassed £1.00bn (€1.15bn; $1.34bn).^1^ Yet eight randomised controlled trials have shown with notable consistency that ASD provides no short term (12 to 24 months) or medium term (≤5 years) advantage over placebo surgery^5^ ^6^ ^7^ or non-surgical management^8^ ^9^ ^10^ ^11^ ^12^ ^13^ for pain relief, function, or health related quality of life for patients with subacromial pain. Based on this evidence, both a BMJ Rapid Recommendation^14^ and a Cochrane review^15^ issued strong recommendations in 2019 against the use of ASD. Despite this advice, ASD remains endorsed in some specialty society clinical practice guidelines,^16^ including NHS England’s Evidence-Based Interventions,^17^ for patients with persistent symptoms after non-operative treatment.

We conducted a multicentre, randomised, placebo surgery controlled trial to evaluate the long term (10 year) efficacy of ASD compared with placebo surgery in patients with subacromial pain syndrome. In addition, the trial included an exploratory, pragmatic secondary comparison between ASD and exercise therapy as a non-operative alternative. The follow-up results at two and five years are published elsewhere.^5^ ^7^

## Methods

### Study design and oversight

FIMPACT (Finnish Subacromial Impingement Arthroscopy Trial), a multicentre, randomised, placebo surgery controlled, superiority trial, was designed to evaluate the efficacy of ASD compared with placebo surgery (diagnostic arthroscopy) in patients with subacromial pain. The trial also included exercise therapy as a non-operative treatment alternative to facilitate a pragmatic comparison between ASD and exercise therapy. Clinical follow-up assessments were carried out at two, five, and 10 years. The protocol was approved by the institutional review board of the Pirkanmaa Hospital District. Reporting follows the consolidated standards of reporting trials (CONSORT) guidelines.^18^ All participants gave written informed consent. On entering the study, participants were informed that they might undergo diagnostic arthroscopy, in which case subacromial decompression would not be performed. They were also informed of the possibility of unblinding or treatment conversion if debilitating symptoms persisted. No prespecified criteria were used to determine “inadequate” relief of symptoms. The participants in the two primary study groups (ASD and placebo surgery) as well as individuals who collected the data were unaware of the study group assignments. Details of the trial protocol are published elsewhere.^19^

### Enrolment and randomisation

We screened patients with shoulder pain who had been referred from primary care to an orthopaedic department for surgical consultation. All participants had magnetic resonance imaging with intra-articular contrast to exclude any other shoulder conditions. Participants were considered eligible for the study if they were men or women aged 35-65 years, had experienced subacromial pain for longer than three months with no relief from non-operative intervention (physiotherapy, non-steroidal anti-inflammatory drugs, corticosteroid injections, and rest), had experienced pain provoked by abduction and arm lifting (positive arc sign), had a positive impingement test result (temporary relief of pain from subacromial injection of lidocaine), had experienced pain during at least two out of three isometric tests (abduction 0° and 30° or external rotation), had provided informed consent, and had the ability to speak, understand, and read in the language of the clinical site. We excluded patients with full thickness tear of the rotator cuff tendons diagnosed on clinical examination (marked weakness in any of the examined muscles) or magnetic resonance imaging with intra-articular contrast; osteoarthritis of the glenohumeral or acromioclavicular joint, or both, diagnosed during clinical examination or by radiography; substantial calcific deposits in the rotator cuff tendons found during preoperative imaging; previous surgery on the affected shoulder; evidence of shoulder instability (positive apprehension or positive sulcus sign); symptomatic cervical spine disease; and a alcohol dependency, drug misuse, psychological problems, or psychiatric problems that might invalidate informed consent.

Recruitment started on 1 February 2005, before the International Committee of Medical Journal Editors’ requirement for prospective trial registration (1 July 2005). The trial was retrospectively registered in 2007 when the FIMPACT team became aware that this requirement also applied to investigator initiated trials.

We used twofold sequential randomisation, with participants first randomised to surgery or exercise therapy in a 2:1 ratio during the baseline appointment. Participants randomised to exercise therapy started standardised physiotherapy within two weeks of the baseline appointment, and those randomised to surgery were scheduled for the procedure within three months of the first randomisation. On the day of surgery, participants underwent diagnostic arthroscopy. If no pathology requiring surgical treatment beyond ASD was identified, they were randomised to either arthroscopic subacromial decompression (ASD) or placebo surgery in a 1:1 ratio. Only the orthopaedic surgeons and operating theatre staff were aware of the surgical group assignment and they did not participate in further treatment or follow-up of participants.

Randomisation was carried out using sequentially numbered sealed opaque envelopes. A statistician with no involvement in the clinical care of trial participants prepared separate randomisation lists for each centre using varied block size.

### Interventions

#### Placebo surgery (diagnostic arthroscopy)

We carried out arthroscopic examination of the glenohumeral joint and subacromial space and assessed rotator cuff integrity using standard posterior and lateral portals and a 4 mm arthroscope, with the participant under general anaesthesia and usually supplemented with an interscalene brachial plexus block. If the rotator cuff tendons could not be visualised, we either bluntly opened the subacromial bursa with a trocar or minimally resected the site. If arthroscopic examination revealed any pathology requiring intervention other than ASD, we excluded the patient from the trial.

Once eligibility was confirmed, participants were randomly assigned to either ASD or placebo surgery. For those allocated to the placebo surgery group, the operation was terminated. To ensure concealment of the allocation from participants and the staff other than those in the operating theatre, all participants remained in the operating theatre for the time required to perform ASD.

#### Arthroscopic subacromial decompression

For participants allocated to ASD after diagnostic arthroscopy, we continued the surgery by performing a standard ASD procedure—a subacromial bursectomy and resection of bony spurs and the projecting anterolateral undersurface of the acromion with a shaver, burr, or electrocoagulation, or combination.^20^

In all surgically treated participants (ASD and placebo surgery groups), postoperative rehabilitation was identical. It consisted of one outpatient visit to a study physiotherapist, blind to the group assignment, for guidance and instructions for home exercises.

#### Exercise therapy

Participants allocated to exercise therapy completed a supervised, progressive, individually designed programme. This programme comprised 15 visits to specially trained physiotherapists who supervised a standardised home exercise protocol (see supplementary appendix for details).

### Clinical management of participants

After being randomised and allocated treatment, participants with persistent or severe symptoms at any point during follow-up were re-evaluated at the study clinic. Based on the clinical assessment, and imaging when appropriate, these participants were managed pragmatically with the treatment deemed most appropriate, such as extended physiotherapy, ASD, or another surgical procedure. In the two surgical groups, unblinding to treatment allocation occurred only if no alternative explanation for the symptoms could be found and ASD was being considered. In the exercise therapy group, unblinding was not relevant, but participants could still be considered for surgery if clinically indicated.

### Outcome measures

The characteristic symptoms of subacromial pain syndrome are shoulder pain at rest, during the night, and on arm elevation. We therefore selected shoulder pain at rest and on arm activity as our primary outcome measures. Pain was assessed using a visual analogue scale (VAS) ranging from 0 (no pain) to 100 (worst pain imaginable). Participants marked their pain on a 10 cm horizontal line, with scores (0-100) derived by measuring the distance in millimetres from the “no pain” anchor to the mark. The minimal important difference (MID) was set at 15 points.^21^

Secondary outcomes included functional assessments using the Constant-Murley score and the simple shoulder test, along with health related quality of life measures, including the SF-36^22^ and 15D^23^ instruments. The MID was set at 17 points as for the Constant-Murley score^24^ and 2 points for the simple shoulder test.^25^ Patients’ global satisfaction with treatment was assessed with the question: “Are you satisfied with the treatment you have received?” Responses were recorded using a VAS ranging from 0 (completely disappointed) to 100 (very satisfied). Patients’ satisfaction with the treatment outcome was assessed with the question: “How satisfied are you with the outcome of your treatment?” Responses were recorded on a five item Likert scale. We categorised participants as responders if they answered: “Very satisfied” or “Satisfied.”

Questionnaires were administered at baseline; three, six, 12, and 24 months; and five and 10 years after randomisation. We assessed adverse events at each follow-up time point, defined as any untoward medical event, regardless of its established association with the treatment. Serious adverse events were those resulting in considerable or lasting disability, need for hospital admission or extended care, a life threatening risk, or death. We only report adverse events considered directly related to the treatment.

### Statistical analysis

We calculated that a sample size of 204 participants (68 per arm; two sided α=0.05, β=90%) would be needed to detect a difference of at least the MID (15 points^21^) in the two primary outcomes between the ASD and placebo surgery groups. Considering the stringent power threshold, we reserved only 3% surplus for potential loss to follow-up and crossovers and accordingly set the recruitment target at 70 participants in each treatment group.

The trial was designed to determine whether ASD was superior to placebo surgery in reducing shoulder pain during rest and arm activity (primary outcomes) over a 10 year follow-up period. We also included a pragmatic comparison between ASD and exercise therapy, using the same two primary outcomes. An independent statistician performed all analyses according to the predefined statistical analysis plan (see supplementary appendix).

We quantified the treatment effect using an intention-to-treat approach for the ASD versus placebo surgery comparison and a full analysis set for the ASD versus exercise therapy comparison. Participants were analysed according to their original randomisation. Treatment effects were estimated as between group differences in pain scores (VAS), Constant-Murley score, simple shoulder test score, 15D score, and SF-36 score, with associated 95% confidence intervals (CIs), at 10 years after the primary randomisation.

A mixed model for repeated measurements analysis of variance was used with participant as a random factor (time points: three, six, 12, and 24 months, and five and 10 years), the baseline value as a covariate, and identity covariance as the covariance structure. This model accommodates missing values under the missing at random assumption, allowing analysis of unbalanced datasets without imputation. Supplementary table S1 shows the missingness of the outcome data. We used the mixed procedure in Stata to fit the mixed model for repeated measurements, with Satterthwaite’s method used to estimate degrees of freedom.

For categorical variables, we used multilevel mixed effects logistic regression, accounting for repeated measures. These variables included patient reported satisfaction, subjective improvement, the proportions of responders and non-responders based on patients’ satisfaction with the treatment outcome, and the incidence of unblinding, treatment conversions, and reoperations at 10 years.

To safeguard against potential multiplicity effects^26^ in the primary comparison, we required a statistically significant treatment effect for both primary outcome measures. We did not predefine any method for adjustment of CIs for multiple comparisons of secondary outcomes. These results are presented as point estimates with unadjusted CIs, and all secondary analyses were supportive and exploratory or hypothesis generating only.

We carried out two prespecified sensitivity analyses (per protocol and as treated) with the same principles as the intention-to-treat and full analysis set analyses. The per protocol population was the subset of participants in the intention-to-treat population who received the treatment they were randomised to and who did not receive any other treatment—that is, we excluded the participants with a treatment conversion (ASD: n=56, placebo surgery: n=47). The as treated population was defined according to the treatment the participants received—that is, the participants who originally received placebo surgery or exercise therapy but owing to persistent symptoms requested unblinding and subsequently received ASD, were included in the ASD population (ASD: n=79, placebo surgery: n=47). We considered a P value of 0.05 to indicate statistical significance. Stata version 18 (StataCorp, TX) was used for all statistical analyses.

### Patient and public involvement

No patients or members of the public were involved in designing the study nor were they involved in developing plans for recruitment, design, or implementation of the study. No patients were asked to advise on interpretation or writing up of results. After the 10 year follow-up, the individual treatment allocation was revealed to the participants.

## Results

### Characteristics of participants

**Fig 1:**
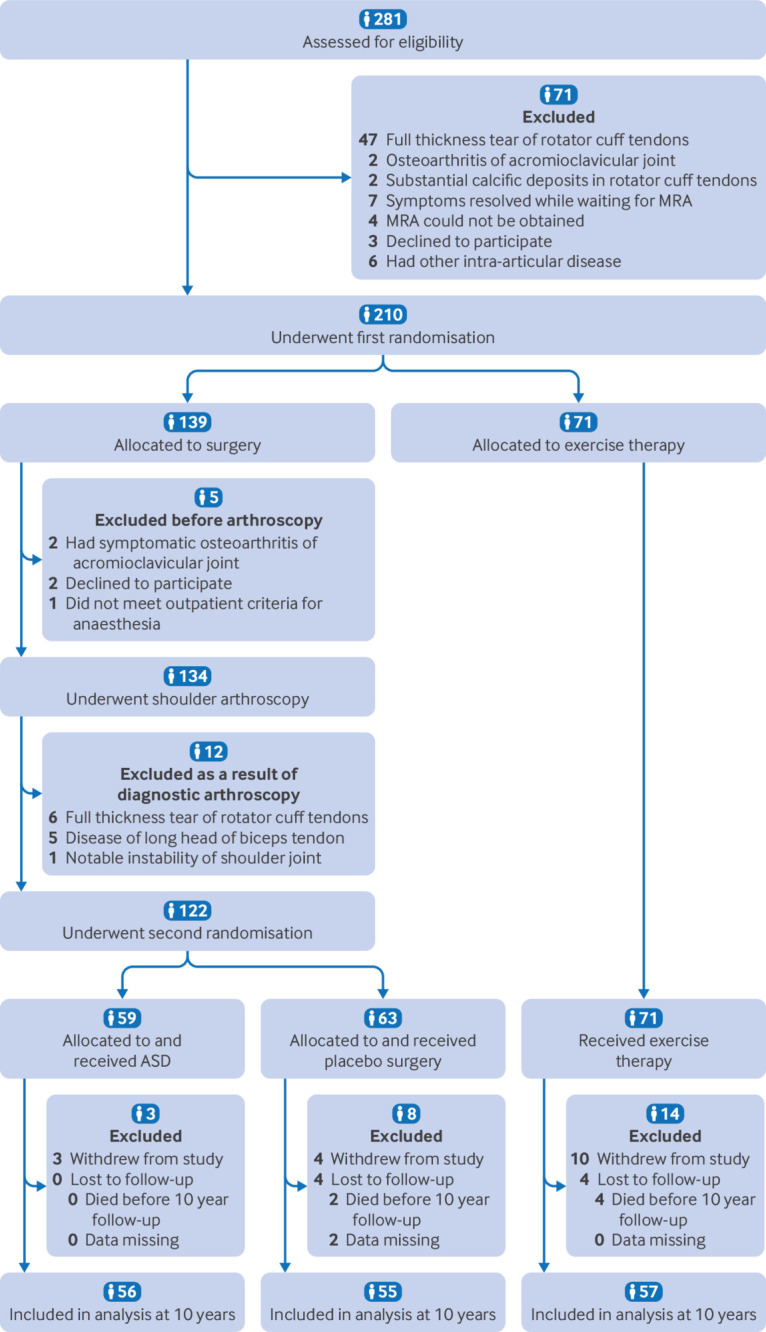
Study flowchart (see supplementary table S2 for full details of unblinding, treatment conversions, and reoperations). ASD=arthroscopic subacromial decompression; MRA=magnetic resonance arthrography

Of the original sample of 210 participants, 56 of 59 participants randomised to ASD (95%), 55 of 63 randomised to placebo surgery (87%), and 57 of 71 randomised to exercise therapy (80%) completed the 10 year follow-up (fig 1).

The study groups were well balanced across all baseline characteristics (table 1). Participants who withdrew from the study (n=17) had comparable baseline primary outcome measures to those who were randomised.

**Table 1:** Baseline characteristics of participants according to study group. Values are mean (standard deviation) unless stated otherwise

Characteristics	ASD (n=59)	Placebo surgery (n=63)	Exercise therapy (n=71)
Age (years)	50.5 (7.3)	50.8 (7.6)	50.4 (6.6)
No (%) women	42 (71)	46 (73)	47 (66)
No (%) with dominant hand affected	35 (59)	36 (57)	46 (65)
Duration of symptoms (months)	18 (14)	18 (19)	22 (23)
No (%) able to work normally with shoulder symptoms	27 (46)	31 (49)	35 (49)
VAS score at rest*	41.3 (25.8)	41.6 (25.5)	41.7 (27.5)
VAS score on arm activity*	71.2 (23.6)	72.3 (21.7)	72.4 (20.8)
Constant-Murley score†	32.2 (15.8)	31.7 (14.0)	35.2 (16.2)
Simple shoulder test score‡	4.9 (2.9)	4.9 (2.9)	4.8 (2.7)
15D score§	0.890 (0.058)	0.891 (0.070)	0.888 (0.078)
SF-36 score physical health; mental health¶	74.3 (12.5); 79.4 (14.2)	74.1 (13.1); 77.9 (16.7)	75.7 (10.1); 75.6 (18.2)

ASD=arthroscopic subacromial decompression; VAS=visual analogue scale.

* Assessed on a 100 mm VAS of 0 to 100. Scores range from 0 (no pain) to 100 (worst pain imaginable).

† Scoring system for various shoulder disorders consisting of objective (range of motion and strength) and subjective (pain assessment, workload, and leisure time activities) measurements. Scores range from 0 and 100, with higher scores indicating better shoulder function.

‡ Based on 12 questions with yes (1) or no (0) response options; maximum score is 12, indicating normal shoulder function; minimum score of 0 points indicates severely diminished shoulder function.

§ Generic health related quality of life instrument comprising 15 dimensions; maximum score is 1 (full health), and minimum score is 0 (death).

¶ Generic health related quality of life instrument to quantify the physical, functional, and psychological aspects of health related quality of life. Consists of 36 questions in eight subscales that assess physical, functional, social, and psychological wellbeing. Score ranges from 0 to 100, with higher scores denoting better health.

### Unblinding

During the 10 year follow-up period, eight of 59 participants in the ASD group and 15 of 63 in the placebo surgery group requested unblinding (P=0.25). Of those unblinded, four in the ASD group and 13 in the placebo surgery group underwent reoperation; two participants in the ASD group and one participant in the placebo surgery group required a third procedure. In addition, one participant in the placebo surgery group underwent rotator cuff repair without previous unblinding. In the exercise therapy group, 19 participants underwent surgery, including four who later required secondary procedures. Supplementary table S2 provides full details.

### Primary outcomes

**Fig 2:**
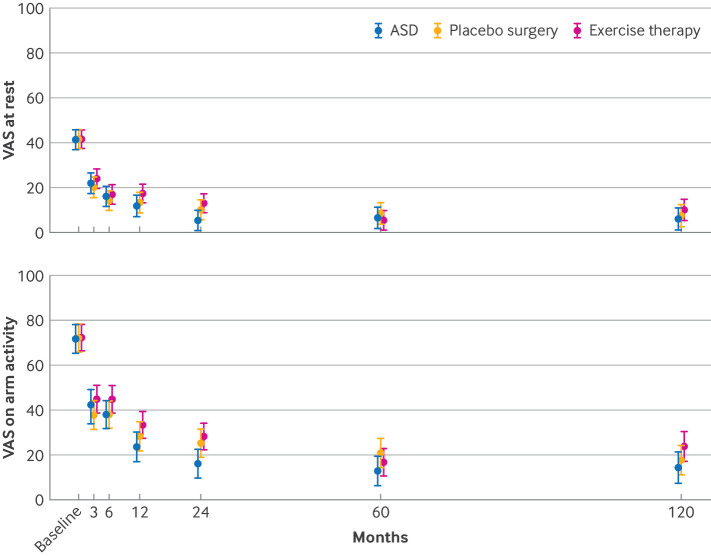
Mean (95% confidence interval) VAS scores for primary outcomes (shoulder pain at rest and on arm activity) by intervention group during 10 year follow-up. Scores range from 0 to 100, with higher scores denoting more severe pain. ASD=arthroscopic subacromial decompression; VAS=visual analogue scale

Noticeable improvements from baseline to 10 years were observed in all three groups for both primary outcomes. The mean changes in shoulder pain were: ASD, 35.5 points for VAS at rest and 57.0 points for VAS on arm activity; placebo surgery, 34.3 points at rest and 54.9 points on arm activity; exercise therapy, 31.9 points at rest and 48.8 points on arm activity (fig 2, table 2). Most of these improvements occurred by the five year follow-up—that is, no additional changes were observed between the five year and 10 year assessments (see supplementary table S5).

**Table 2:** Primary and secondary outcomes of the trial at 10 year follow-up. Values are means (95% confidence intervals) unless stated otherwise

	ASD (n=56)	Placebo surgery* (n=55)	Exercise therapy (n=57)	Between group difference, ASD *v* placebo surgery	Between group difference, ASD *v* exercise therapy
**Primary outcomes**					
VAS score at rest†	5.8 (0.7 to 10.9)	7.3 (2.3 to 12.2)	9.8 (5.0 to 14.6)	−1.5 (−8.6 to 5.6)	−4.0 (−11.0 to 3.0)
VAS score on arm activity†	14.2 (7.2 to 21.2)	17.4 (10.7 to 24.2)	23.6 (16.9 to 30.2)	−3.2 (−13.0 to 6.5)	−9.4 (−19.0 to 0.3)
**Secondary outcomes**					
Constant-Murley score	80.3 (75.5 to 85.2)	81.8 (77.2 to 86.4)	76.2 (71.7 to 80.8)	−1.5 (−8.2 to 5.2)	4.1 (−2.5 to 10.8)
Simple shoulder test score	10.9 (10.2 to 11.5)	10.8 (10.2 to 11.5)	10.6 (10.0 to 11.2)	0.0 (−0.9 to 0.9)	0.3 (−0.6 to 1.2)
15D score	0.898 (0.885 to 0.912)	0.902 (0.888 to 0.916)	0.904 (0.890 to 0.918)	−0.003 (−0.023 to 0.016)	−0.006 (−0.025 to 0.013)
SF-36 score physical health; mental health	82.7 (79.4 to 86.0); 78.5 (75.4 to 81.7)	83.6 (80.2 to 86.9); 80.3 (77.1 to 83.5)	82.7 (79.4 to 86.0); 81.4 (78.2 to 84.5)	−0.8 (−5.6 to 3.9); −1.8 (−6.3 to 2.7)	0.0 (−4.7 to 4.7); −2.8 (−7.3 to 1.6)
Proportion of participants able to return to previous leisure activities‡	0.87 (0.78 to 0.96)	0.94 (0.88 to 1.00)	0.83 (0.73 to 0.93)	−0.07 (−0.18 to 0.04)	0.04 (−0.10 to 0.17)
Proportion of responders§	0.88 (0.79 to 0.97)	0.84 (0.74 to 0.94)	0.84 (0.75 to 0.94)	0.04 (−0.09 to 0.17)	0.03 (−0.10 to 0.16)
Patients’ satisfaction with treatment¶	0.89 (0.84 to 0.95)	0.85 (0.80 to 0.90)	0.88 (0.83 to 0.93)	0.05 (−0.02 to 0.12)	0.02 (−0.05 to 0.09)
No (%) of complications and adverse events**	3 (5)	2 (3)	3 (4)	-	-

ASD=arthroscopic subacromial decompression; VAS=visual analogue scale.

Lower scores indicate the desired (better) treatment outcome in VAS pain score and complications, whereas higher scores indicate the same in all other outcomes. Between group differences may not exactly equal the difference in change in score between the ASD and placebo surgery groups because of the adjustment for baseline imbalance in the mixed effects model.

* Diagnostic arthroscopy.

† Scores range from 0 (no pain) to 100 (worst pain imaginable).

‡ Assessed with the question: “Have you been able to return to your previous leisure activities?” (yes or no).

§ Patients’ satisfaction with the treatment outcome was elicited with a question: “How satisfied are you with the outcome of your treatment?” on a five item scale. Participants who reported very satisfied or satisfied were categorised as responders.

¶ Patients’ global assessment of satisfaction to the treatment was elicited with the question: “Are you satisfied with the treatment you have received?” with VAS scores ranging from 0 (completely disappointed) to 100 (very satisfied).

** Registered complications directly related to the interventions.

For the primary comparison between ASD and placebo surgery, no significant differences were observed for either primary outcome: VAS pain score at rest (mean difference –1.5, 95% CI –8.6 to 5.6) and VAS pain score on arm activity (–3.2, –13.0 to 6.5). In the secondary comparison, ASD also did not differ from exercise therapy: VAS pain score at rest (–4.0, –11.0 to 3.0) and VAS pain score on arm activity (–9.4, –19.0 to 0.3) (fig 2, table 2, supplementary table S3).

### Secondary outcomes

No significant between group differences were observed for any secondary outcomes (table 2, supplementary table S4). Similarly, no statistically significant changes were detected between the five year and 10 year follow-up for any secondary outcome (see supplementary table S5).

### Prespecified sensitivity analyses

The results remained unaltered in the prespecified sensitivity (as treated and per protocol) analyses (see supplementary tables S6 and S7), except for one secondary comparison (per protocol, ASD versus exercise therapy for pain on arm activity), which reached statistical significance but not the prespecified threshold for MID. Importantly, all sensitivity analyses supported the primary comparison between ASD and placebo surgery.

### Complications and adverse events

One participant in the placebo surgery group experienced transient swelling at the injection site of the brachial plexus block. In addition, frozen shoulder developed in three participants in the ASD group, one in the placebo surgery group, and two in the exercise therapy group (table 2). One participant also reported aggravation of low back pain during the exercise therapy regimen. No other complications or adverse events directly attributable to the interventions were observed.

## Discussion

This multicentre, randomised trial found no long term benefit of ASD over placebo surgery in managing subacromial pain. To date, the only other placebo surgery controlled trial on ASD is the Can Shoulder Arthroscopy Work? (CSAW) trial.^6^ Both CSAW and FIMPACT showed no meaningful benefit of ASD over placebo surgery for pain or functional outcomes in the short term (one year for CSAW,^6^ two and five years for FIMPACT^5^ ^7^), a result now confirmed by FIMPACT’s 10 year follow-up as well. Our efficacy design, with a placebo surgery comparator and high participant retention (87%), strengthens the reliability of the effect estimates for the primary comparison of ASD versus placebo surgery.

Two open label trials have compared ASD with exercise therapy beyond five years, with conflicting findings.^27^ ^28^ One of the trials^27^ found no benefit of ASD, whereas the other trial^28^ suggested potential advantages. A 2019 Cochrane review summarised the available evidence as low certainty, suggesting that ASD might improve function but not pain.^15^ However, the authors also acknowledged that the treatment effects at longer follow-up time points may be confounded by increased attrition and reporting bias.^15^ As a result, they plan on updating the review once the long term results of this study become available. These 10 year results help resolve previous uncertainty and reinforce that ASD provides no clinically meaningful benefit over either placebo surgery or exercise therapy.

Earlier publications of FIMPACT^5^ ^7^ have discussed at length various possible limitations of the trial, mostly anticipating or responding to general concerns relating to methodology (see supplementary table S8).

### Conclusions and policy implications

In patients with subacromial pain syndrome, arthroscopic subacromial decompression (ASD) provides no benefit over placebo surgery (or exercise therapy) at 10 years. Across randomised trials, the evidence is notably consistent: ASD offers no short term, medium term, or long term advantage in pain, function, or quality of life, and no trial has identified a subgroup with benefit. Despite this, ASD continues to be performed—a pattern that seems to be typical of medical reversals—sustained by financial incentives, entrenched professional beliefs, and institutional inertia. Based on current evidence, ASD should not be offered outside rigorously designed clinical trials. De-implementation in routine care is warranted: health systems, clinicians, and patients should consider adopting less burdensome treatments for subacromial pain.

**What is already known on this topic**

Despite systematic reviews and meta-analyses of randomised controlled trials consistently showing that arthroscopic subacromial decompression (ASD) versus exercise therapy or placebo surgery provides no short term improvement in shoulder pain, ASD remains one of the most common elective orthopaedic procedures
Evidence for ASD offering long term relief from shoulder pain remains inconsistent—two open label trials with follow-up beyond five years reported conflicting outcomes: one found no benefit and the other found potential advantages

**What this study adds**

In this placebo surgery controlled Finnish Subacromial Impingement Arthroscopy Trial (FIMPACT) of patients with subacromial pain, arthroscopic subacromial decompression (ASD) showed no additional benefit over placebo surgery or exercise therapy at 10 years
All treatment groups showed substantial and sustained improvement in pain and shoulder function during follow-up
These results indicate that ASD does not provide meaningful clinical advantage in the long term

## Data Availability

The code used to analyse the data in the paper can be found in the supplemental files. The data underlying the findings in this paper are openly and publicly available at https://doi.org/10.23729/fd-d323a34b-f698-3bc6-b38d-9c93aeadbe74. If you encounter problems accessing the data, please contact the corresponding author.

## Web extra

Extra material supplied by authors

Supplementary information: FIMPACT Investigators, tables S1-S8, exercise therapy protocol, and statistical analysis plan

## Notes

Contributors: MP and TJ were co-principal investigators. KK and MB shared authorship. ST, TLNJ, and MP provided equal contributions. MP, AM, ST, and TJ conceived and designed the study. JI also contributed to the design. KK, JK, IS, and VS recruited most of the patients. The FIMPACT Investigators followed up the patients. MP, KK, ST, and TJ collected the data. ST designed the database and cleaned the data. ST, KK, TJ, and TC contributed to the study analysis plan and data analysis. KK, MB, ST, TC, TJ, and MP participated in the analysis and interpretation of the data. KK, ST, MP, MB, TC, and TJ drafted the manuscript. All authors contributed to final data interpretation and contributed to and approved the final draft of the manuscript. MP and TJ obtained funding. MP is the guarantor. The corresponding author attests that the writing committee and all listed collaborators meet authorship criteria and that no others meeting the criteria have been omitted.

Transparency: The lead author (MP) affirms that the manuscript is an honest, accurate, and transparent account of the study being reported; that no important aspects of the study have been omitted; and that any discrepancies from the study as planned have been explained.

Dissemination to participants and related patient and public communities: Upon publication of the trial results, a lay summary will be distributed to recruiting centres for sharing with participants. Results will also be disseminated to the wider public through press releases, traditional media, social media channels, and conference presentations.

Provenance and peer review: Not commissioned; externally peer reviewed.
