# Supplementary material for: Arthroscopic subacromial decompression versus placebo surgery for subacromial pain syndrome: 10 year follow-up of the FIMPACT randomised, placebo surgery controlled trial
Source: BMJ. 2025 Dec 2;391:e086201. doi: 10.1136/bmj-2025-086201 (PMC12670246; doi:10.1136/bmj-2025-086201)
Supplement: Supplementary file 1 — Supplementary information: FIMPACT Investigators, tables S1-S8, exercise therapy protocol, and statistical analysis plan [file kank086201.ww.pdf]

Supplementary Appendix

This appendix has been provided by the authors to offer readers additional information about their work.

Table of Contents

FIMPACT Investigators 3

Table S1 Baseline characteristics of the participants according to study group 5

Table S2 Trajectories of the primary outcomes at 33, 6, 12, 24 months, 5 years and 10 years 6

Table S3 Trajectories of the secondary outcomes at 3, 6, 12, 24 months, 5 years and 10 years 8

Table S4 Changes Observed from the 5-Year to the 10-Year Follow-Up 11

Table S5 Sensitivity analyses of the primary and secondary outcomes concerning the primary comparison (ASD vs. Placebo surgery) 12

Table S6 Sensitivity analyses of the primary and secondary outcomes concerning the secondary comparison (ASD vs. ET) 13

Table S7 Unblindings, treatment conversions, and reoperations 14

Table S8 Frequency of missing data at 3, 6, 12, 24 months, 5 years and 10 years 15

Table S9 Key Concerns Regarding Prior Publications of the FIMPACT Trial 16

Exercise Therapy (ET) Protocol 19

Statistical Analysis Plan for the FIMPACT Trial at the 10-year Follow-Up 35

Complete list of FIMPACT investigators

Authors (*to be indexed on PubMed*):

Kari Kanto, MD, PhD[1,2], Mathias Bäck, MD[1,3], Thomas Ibounig, MD[1,3], Robert Björkenheim, MD[1,3], Antti Malmivaara, MD, PhD[4,5], Tomasz Czuba, PhD[6], Jari Inkinen, PT[7], Juha Kalske, MD[8], Vesa Savolainen, MD, PhD[9], Ilkka Sinisaari, MD, PhD[10], Pirjo Toivonen, PT[1,3], Simo Taimela, MD, PhD[1,3], Teppo L N Järvinen, MD, PhD[1,3], Mika Paavola, MD, PhD[1,3]

Collaborators *(to be indexed on PubMed*):

Jonas Ranstam, PhD[11], Jarkko Pajarinen, MD, PhD[10]; Sikri Tukiainen, MD[12]; Kalevi Hietaniemi, MD, PhD[3]; Vesa Lepola, MD, PhD[13]; Jyrki Salmenkivi, MD[3]; Mikko Salmela, MD, PhD[3]; Timo Järvelä, MD, PhD[14]; Janne Lehtinen, MD, PhD[2]; Ville Haapamäki, MD, PhD[15]; Heikki Kolehmainen, MD, PhD[16]; Mikael Salmela, MD[17]; Tarja Kunnala, PT[8]; Sami Niskanen, PT[12]; Hanna-Mari Laiho, PT[2]; Leena Kangas-Viri, PT[2]; Leena Caravitis, PT[1,3]; Sanna Hokkanen RN[3]; Marketta Rautanen, RN[2]; Sari Karesvuori, RN[8]; Soile Lindholm, RN[3]; Eero Hölli, PT[12]; Lena Laine, PT[12] and Esa Läärä, Professor[18]

[1] Finnish Centre for Evidence-Based Orthopaedics (FICEBO), University of Helsinki, Helsinki, Finland

[2] Department of Orthopaedics and Traumatology, Tampere University Hospital, Tampere, Finland

[3] Department of Orthopaedics and Traumatology, Helsinki University Hospital, Haartmaninkatu 4, PO Box 320, 00029 HUS, Helsinki, Finland

[4] National Institute for Health and Welfare, Centre for Health and Social Economics, Helsinki, Finland

[5] Orton Orthopaedic hospital, Helsinki, Finland

[6] Department of Molecular and Clinical Medicine, University of Gothenburg, Gothenburg, Sweden

[7] Physiotherapy centre Fysios Mehiläinen, Tampere, Finland

[8] Department of Orthopaedics and Traumatology, Helsinki University Hospital, Jorvi hospital, Espoo, Finland

[9] Pihlajalinna Hospital, Helsinki, Finland

[10] Terveystalo Hospital, Helsinki, Finland

[11] Mdas AB, Ystad, Sweden

[12] Mehiläinen Hospital, Helsinki, Finland

[13] Pihlajalinna Hospital, Tampere, Finland

[14] Mehiläinen Hospital, Tampere, Finland

[15] HUS Medical Imaging Centre, Helsinki, Finland

[16] Department of Radiology, Tampere University Hospital, TAYS Hatanpää, Tampere, Finland

[17] Terveystalo Hospital, Tampere, Finland

[18] Research Unit of Mathematical Sciences, University of Oulu, Oulu, Finland

Contributions:

Writing Committee: KK, MB, ST, TJ and MP.

Steering Committee: MP, ST, AM, and TJ.

Clinical Sites:

Helsinki University Hospital, Jorvi Hospital, Helsinki:

Clinical Site Investigators: Kalevi Hietaniemi, Juha Kalske, Vesa Lepola, Jyrki Salmenkivi and Sikri Tukiainen.

Physiotherapy (follow-up examinations): Tarja Kunnala

Imaging: Ville Haapamäki

Research coordinators: Leena Caravitis, Sari Karesvuori

Helsinki University Hospital, Herttoniemi Hospital, Helsinki:

Clinical Site Investigators: Jarkko Pajarinen, Mikko Salmela, Vesa Savolainen and Ilkka Sinisaari

Physiotherapy (follow-up examinations): Sami Niskanen

Research coordinators: Leena Caravitis and Soile Lindholm

Tampere University Hospital, Tampere:

Clinical Site Investigators: Timo Järvelä, Kari Kanto, Janne Lehtinen and Mikael Salmela

Imaging: Heikki Kolehmainen

Physiotherapy (follow-up examinations): Hanna-Mari Laiho and Leena Kangas-Viri

Research coordinators: Pirjo Toivonen, Marketta Rautanen.

Independent Physiotherapists:

Jari Inkinen, Eero Hölli, Lena Laine

FIMPACT Methods Center:

Pirjo Toivonen (Project management), Mathias Bäck (Data management) and Esa Läärä (Randomisation).

Table S1 Baseline characteristics of the participants according to study group			
**Characteristics**	**ASD (n=59)**	**Placebo surgery (n=63)**	**ET (n=71)**
Age, years	50.5 (7.3)	50.8 (7.6)	50.4 (6.6)
Female, n (%)	42 (71)	46 (73)	47 (66)
Dominant hand affected, n (%)	35 (59)	36 (57)	46 (65)
Duration of symptoms, months	18 (14)	18 (19)	22 (23)
Able to work normally regardless of shoulder symptoms, n (%)	27 (46)	31 (49)	35 (49)
Visual analogue scale score, at rest*	41.3 (25.8)	41.6 (25.5)	41.7 (27.5)
Visual analogue scale score, on arm activity*	71.2 (23.6)	72.3 (21.7)	72.4 (20.8)
Constant-Murley score†	32.2 (15.8)	31.7 (14.0)	35.2 (16.2)
Simple shoulder test score‡	4.9 (2.9)	4.9 (2.9)	4.8 (2.7)
15D score§	0.890 (0.058)	0.891 (0.070)	0.888 (0.078)
SF-36 score ¶physical health	74.3 (12.5)	74.1 (13.1)	75.7 (10.1)
mental health	79.4 (14.2)	77.9 (16.7)	75.6 (18.2)

ASD = arthroscopic subacromial decompression; ET = exercise therapy

Data are presented as mean (SD) unless otherwise indicated.

*Shoulder pain at rest and at activity was assessed on a 100 mm Visual analogue scale (VAS) of 0 to 100, with 0 denoting no pain and 100 denoting extreme pain.

†Scoring system for evaluation of various shoulder disorders consisting of both objective (range of motion and strength) and subjective measurements (pain assessment, workload, and leisure time activities), summarised in a score between 0 and 100; higher score indicates better shoulder function.

‡Based on 12 questions with yes (1) or no (0) response options; maximum score is 12, indicating normal shoulder function; minimum score of 0 points indicates severely diminished shoulder function.

§Generic health related quality of life instrument comprising 15 dimensions; maximum score is 1 (full health), and minimum score is 0 (death).

¶Generic health related quality of life instrument to quantify the physical, functional, and psychological aspects of health-related quality of life. It consists of 36 questions in eight subscales that assess physical, functional, social, and psychological well-being. Score ranges from 0 to 100, with higher scores indicating better health.

Table S2 Trajectories of the primary outcomes at 3, 6, 12, 24 months, 5 and 10 years								
**Outcomes**	**ASD**	**n**	**Placebo surgery**	**n**	**ET**	**n**	**Between-group difference, ASD vs. Placebo surgery**	**Between-group difference, ASD vs. ET**
**3 months**								
Pain VAS at rest	21.6 (17.0 to 26.3)	54	19.8 (15.0 to 24.3)	55	23.8 (19.4 to 28.3)	62	1.9 (-4.6 to 8.4)	-2.1 (-8.7 to 4.4)
Pain VAS on arm activity	42.1 (35.4 to 48.8)	52	37.5 (31.0 to 44.0)	55	44.7 (38.7 to 50.8)	62	4.6 (-4.8 to 14.0)	-2.4 (-11.5 to 7.0)
**6 months**								
Pain VAS at rest	15.9 (11.3 to 20.5)	59	13.9 (9.4 to 18.5)	61	16.9 (12.6 to 21.2)	68	1.9 (-4.3 to 8.2)	-0.9 (-7.2 to 5.4)
Pain VAS on arm activity	37.9 (31.5 to 44.4)	59	37.7 (31.5 to 43.9)	61	44.6 (38.7 to 50.5)	68	0.1 (-8.7 to 9.0)	-6.5 (-15.1 to 2.1)
**12 months**								
Pain VAS at rest	11.6 (7.0 to 16.2)	55	13.9 (9.6 to 18.3)	56	17.1 (12.8 to 21.5)	65	-1.5 (-8.0 to 4.9)	-5.5 (-11.9 to 0.9)
Pain VAS on arm activity	23.3 (16.7 to 29.8)	55	28.0 (21.5 to 34.4)	56	33.1 (27.1 to 39.1)	64	-4.7 (-13.8 to 4.5)	-9.6 (-18.4 to -0.8)
**24 months**								
Pain VAS at rest	5.2 (0.8 to 9.6)	59	9.8 (5.2 to 14.2)	59	12.8 (8.5 to 17.1)	68	-4.6 (-10.8 to 1.7)	-7.5 (-13.8 to -1.2)
Pain VAS on arm activity	15.8 (9.5 to 22.1)	59	24.8 (18.5 to 31.1)	59	28.1 (22.2 to 34.0)	68	-9.0 (-17.9 to 0)	-12.1 (-20.7 to -3.5)
**5 years**								
Pain VAS at rest	6.2 (1.6 to 10.8)	54	8.2 (3.5 to 12.8)	54	5.3 (0.8 to 9.8)	68	-2.0 (-8.5 to 4.6)	-1.0 (-5.6 to 7.6)
Pain VAS on arm activity	12.4 (5.8 to 19.0)	54	20.4 (13.8 to 26.9)	54	16.5 (10.3 to 22.6)	68	-8.0 (-17.3 to 1.3)	-3.9 (-12.8 to 5.1)
**10 years**								
Pain VAS at rest	5.8 (0.7 to 10.9)	48	7.3 (2.3 to 12.2)	51	9.8 (5.0 to 14.6)	55	-1.5 (-8.6 to 5.6)	-4.0 (-11.0 to 3.0)

Pain VAS on arm activity	14.2 (7.2 to 21.2)	48	17.4 (10.7 to 24.2)	52	23.6 (16.9 to 30.2)	55	-3.2 (-13.0 to 6.5)	-9.4 (-19.0 to 0.3)

ASD = arthroscopic subacromial decompression; ET = exercise therapy

Pain VAS scores at rest and on arm activity range from 0 (no pain) to 100 (worst pain imaginable).

The table shows the mean values and between-group differences in VAS pain scores at the 3, 6, 12, 24-months, 5- and 10-year follow-ups with 95% confidence intervals. The trajectories were estimated using a mixed-effects model repeated measures (MMRM) regression with adjustment for baseline imbalance. N denotes the number of subjects.

The between-group differences may not exactly equal the differences in change in the scores between groups because of the adjustment for baseline imbalance in the MMRM analyses. A negative between-group difference means that the participants in the ASD group reported a lower pain level.

Table S3 Trajectories of the secondary outcomes at 3, 6, 12, 24 months, 5 and 10 years								
**Outcomes**	**ASD**	**n**	**Placebo surgery**	**n**	**ET**	**n**	**Between-group difference ASD vs. Placebo surgery**	**Between-group difference ASD vs. ET**
**3 months**								
Constant-Murley score	N/A		N/A		N/A		N/A	N/A
SST	N/A		N/A		N/A		N/A	N/A
Satisfaction to the treatment (0-100 VAS)	76.9 (71.9 to 82.0)	57	81.4 (76.3 to 86.4)	57	79.3 (74.5 to 84.0)	67	-4.5 (-11.6 to 2.7)	-2.3 (-9.3 to 4.6)
15D	0.909 (0.895 to 0.923)	57	0.918 (0.904 to 0.932)	57	0.900 (0.887 to 0.913)	65	-0.009 (-0.029 to 0.010)	0.09 (-0.010 to 0.027)
SF-36 score<br>Physical health<br>Mental health	81.6 (78.3 to 84.8)<br>79.8 (77.0 to 82.7)	58<br>58	83.2 (79.9 to 86.5)<br>83.2 (80.3 to 86.1)	57<br>57	81.3 (78.5 to 84.0)<br>80.2 (77.3 to 83.2)	66<br>67	-1.6 (-6.2 to 3.0)<br>-3.3 (-7.4 to 0.8)	0.6 (-3.5 to 4.6)<br>-1.1 (-5.4 to 3.2)
**6 months**								
Constant-Murley score	58.2 (54.0 to 62.4)	59	64.4 (60.2 to 68.5)	61	58.3 (54.3 to 62.3)	68	-6.1 (-12.1 to 0.2)	0.8 (-5.0 to 6.7)
SST	7.9 (7.4 to 8.5)	59	8.5 (7.9 to 9.1)	61	7.9 (7.4 to 8.5)	68	-0.5 (-1.4 to 0.3)	0.0 (-0.80 to 0.80)
Satisfaction to the treatment (0-100 VAS)	75.1 (70.1 to 80.2)	56	79.7 (74.7 to 84.7)	58	75.2 (70.4 to 80.0)	66	-4.5 (-11.7 to 2.6)	-0.0 (-7.1 to 7.0)
15D	0.915 (0.901 to 0.929)	56	0.922 (0.909 to 0.936)	58	0.892 (0.880 to 0.905)	66	-0.007 (-0.027 to 0.012)	0.022 (0.004 to 0.041)
SF-36 score<br>Physical health<br>Mental health	84.9 (81.6 to 88.1)<br>80.6 (77.7 to 83.5)	57<br>57	84.7 (81.5 to 88.0)<br>82.3 (79.4 to 85.1)	58<br>59	81.3 (78.6 to 84.1)<br>80.4 (77.4 to 83.3)	67<br>67	0.1 (-4.5 to 4.8)<br>-1.6 (-5.7 to 2.5)	3.8 (-0.2 to 7.8)<br>-0.5 (-4.8 to 3.8)
**12 months**								
Constant-Murley score	N/A		N/A		N/A		N/A	N/A
SST	N/A		N/A		N/A		N/A	N/A
Satisfaction to the treatment (0-100 VAS)	82.9 (77.8 to 88.0)	55	85.9 (80.7 to 91.0)	54	78.0 (73.2 to 82.9)	64	-2.9 (-10.2 to 4.3)	4.9 (-2.2 to 12.0)

15D	0.916 (0.902 to 0.930)	56	0.929 (0.915 to 0.943)	55	0.909 (0.896 to 0.921)	64	-0.013 (-0.033 to 0.007)	0.007 (-0.012 to 0.026)
SF-36 score<br>Physical health<br>Mental health	86.9 (83.6 to 90.1)<br>77.0 (74.1 to 80.0)	56<br>55	85.3 (82.0 to 88.6)<br>85.2 (82.2 to 88.2)	56<br>57	86.5 (83.6 to 89.3)<br>81.8 (78.7 to 84.8)	64<br>64	1.5 (-3.2 to 6.2)<br>-8.2 (-12.4 to 4.0)	0.6 (-3.5 to 4.7)<br>-5.5 (-9.9 to 1.1)
**24 months**								
Constant-Murley score	78.0 (73.7 to 82.3)	58	73.8 (69.6 to 78.0)	59	71.3 (67.3 to 75.4)	65	4.2 (-1.8 to 10.2)	7.5 (1.6 to 13.4)
SST	10.4 (9.8 to 11.0)	58	9.9 (9.3 to 10.5)	59	9.7 (9.1 to 10.2)	66	0.4 (-0.4 to 1.3)	0.7 (-0.1 to 1.5)
Satisfaction to the treatment (0-100 VAS)	88.2 (83.2 to 93.3)	58	87.4 (82.4 to 92.5)	56	84.8 (80.1 to 89.6)	67	0.8 (-6.3 to 7.9)	3.4 (-3.5 to 10.4)
15-D	0.918 (0.904 to 0.931)	58	0.921 (0.908 to 0.935)	58	0.914 (0.901 to 0.927)	68	-0.004 (-0.023 to 0.016)	0.004 (-0.015 to 0.022)
SF-36 score<br>Physical health<br>Mental health	87.6 (84.4 to 90.9)<br>77.9 (75.0 to 80.8)	58<br>57	86.1 (82.9 to 89.4)<br>82.6 (79.7 to 85.5)	58<br>59	87.3 (84.5 to 90.0)<br>82.8 (79.8 to 85.7)	68<br>68	1.5 (-3.1 to 6.1)<br>-4.7 (-8.8 to 0.6)	0.6 (-3.4 to 4.6)<br>-5.6 (-9.9 to -1.3)
**5 years**								
Constant-Murley score	82.8 (78.4 to 87.3)	58	75.7 (71.3 to 80.1)	59	79.8 (75.6 to 88.1)	65	7.1 (-0.9 to 13.4)	3.9 (2.2 to 10.0)
SST	10.7 (10.1 to 11.3)	58	10.3 (9.7 to 11.0)	59	10.7 (10.1 to 11.2)	66	0.3 (-0.5 to 1.2)	0.0 (-0.9 to 0.8)
Satisfaction to the treatment (0-100 VAS)	89.7 (84.5 to 94.9)	58	85.7 (80.6 to 90.8)	56	86.8 (81.8 to 91.7)	67	4.0 (-3.2 to 11.3)	3.0 (-4.3 to 10.2)
15D	0.903 (0.888 to 0.917)	58	0.912 (0.898 to 0.927)	58	0.916 (0.903 to 0.929)	68	-0.009 (-0.030 to 0.010)	-0.013 (-0.033 to 0.006)
SF-36 score<br>Physical health<br>Mental health	84.0 (80.6 to 87.4)<br>79.9 (76.9 to 82.9)	58<br>57	85.4 (82.0 to 88.8)<br>80.1 (77.0 to 83.2)	57<br>57	87.5 (84.7 to 90.4)<br>81.9 (78.8 to 84.9)	62<br>62	-1.4 (-6.1 to 3.4)<br>-0.2 (-4.5 to 4.1)	-3.3 (-7.4 to 0.9)<br>-2.7 (-7.2 to 1.8)
**10 years**								
Constant-Murley score	80.3 (75.5 to 85.2)	45	81.8 (77.2 to 86.4)	51	76.2 (71.7 to 80.8)	53	-1.5 (-8.2 to 5.2)	4.1 (-2.5 to 10.8)
SST	10.9 (10.2 to 11.5)	48	10.8 (10.2 to 11.5)	52	10.6 (10.0 to 11.2)	55	0.02 (-0.9 to 0.9)	0.3 (-0.6 to 1.2)
Satisfaction to the treatment (0-100 VAS)	89.5 (84.4 to 94.5)	56	84.7 (79.6 to 89.8)	54	87.6 (82.6 to 92.6)	57	4.7 (-2.5 to 12.0)	1.9 (-5.2 to 9.0)
15D	0.898 (0.885 to 0.912)	56	0.902 (0.888 to 0.916)	53	0.904 (0.890 to 0.918)	56	-0.003 (-0.023 to 0.016)	-0.006 (-0.025 to 0.013)

SF-36 score								
Physical health	82.7 (79.4 to 86.0)	56	83.6 (80.2 to 86.9)	54	82.7 (79.4 to 86.0)	57	-0.8 (-5.6 to 3.9)	0.0 (-4.7 to 4.7)
Mental health	78.5 (75.4 to 81.7)	56	80.3 (77.1 to 83.5)	54	81.4 (78.2 to 84.5)	57	-1.8 (-6.3 to 2.7)	-2.8 (-7.3 to 1.6)

ASD = arthroscopic subacromial decompression; ET = exercise therapy

The table shows the mean values and between-group differences in VAS pain scores at the 3, 6, 12, 24-months, 5- and 10-year follow-ups with 95% confidence intervals. The trajectories were estimated using a mixed-effects model repeated measures (MMRM) regression with adjustment for baseline imbalance. N denotes the number of subjects.

The between-group differences may not exactly equal the differences in change in the scores between groups because of the adjustment for baseline imbalance in the MMRM analyses. N/A = not applicable. For all variables, a higher score indicates a better treatment outcome.

Table S4 Changes observed from the 5-year to the 10-year follow-up *			
	**ASD**	**Placebo surgery**	**ET**
**Primary outcomes**			
Vas at rest†	-0.41 (-7.08 to 6.26)	-0.88 (-7.45 to 5.69)	4.64 (-1.64 to 10.92)
Vas on arm activity†	1.79 (-6.92 to 10.49)	-3.15 (-11.66 to 5.36)	7.17 (-1.02 to 15.35)
**Secondary outcomes**			
Constant-Murley score	-3.04 (-9.19 to 3.12)	5.70 (-0.24 to 11.63)	-3.67 (-9.46 to 2.12)
Simple shoulder test score	0.18 (-0.69 to 1.05)	0.51 (-0.33 to 1.36)	-0.08 (-0.89 to 0.73)
15D score	-0.00 (-0.02 to 0.01)	-0.01 (-0.03 to 0.01)	-0.01 (-0.03 to 0.00)
SF-36 score physical health<br>mental health	-1.53 (-5.62 to 2.56)<br>-0.81 (-4.75 to 3.13)	-2.02 (-6.16 to 2.11)<br>0.77 (-3.27 to 4.80)	-4.72 (-8.68 to -0.75)<br>-0.67 (-4.49 to 3.14)
Proportion of participants able to return to previous leisure activities‡	-4.46 (-14.33 to 5.41)	8.23 (-1.78 to 18.24)	3.60 (-7.99 to 15.18)
Proportion of responders§	-0.01 (-11.14 to 11.12)	2.34 (-10.03 to 14.71)	6.18 (-6.14 to 18.49)
Patients' satisfaction with treatment**	-0.12 (-6.06 to 5.82)	-0.68 (-6.60 to 5.23)	0.71 (-5.00 to 6.42)

ASD = arthroscopic subacromial decompression; ET = exercise therapy

*Values are means (10 year - 5 year values) with 95% confidence intervals unless otherwise indicated. A lower score indicates the desired (better) treatment outcome in pain VAS score and complications, while a higher score indicates the same in all other outcomes. Between-group differences may not exactly equal the difference in changes in score between the ASD and placebo-surgery groups because of the adjustment for baseline imbalance in the mixed-effects model.

†Pain VAS scores at rest and on arm activity range from 0 (no pain) to 100 (worst pain imaginable).

‡Participants ability to return to previous leisure activities was assessed with the following question: "Have you been able to return to your previous leisure activities?" ("yes" or "no").

§Patients' satisfaction with the treatment outcome was elicited with a question: "How satisfied are you with the outcome of your treatment?" on a 5-item scale. Participants who reported very satisfied or satisfied were categorised as "Responders".

**Patients' global assessment of satisfaction to the treatment was elicited with this question: "Are you satisfied with the treatment you have received?" We used a VAS scale ranging from 0 (completely disappointed) to 100 (very satisfied).

Table S5 Sensitivity analyses of the primary and secondary outcomes concerning the primary comparison (ASD vs. Placebo surgery)

The table shows the between-group differences at the 10-year follow up. The estimated effect indicates the mean difference, ASD minus Placebo surgery. The analyses were carried out using a mixed model repeated measures (MMRM) regression with adjustment for baseline imbalance.

Variable	ITT<br>Estimated effect (95% CI)*	P value	Per protocol<br>Estimated effect (95% CI)†	P value	As treated<br>Estimated effect (95% CI)‡	P value
Pain VAS at rest	-1.5 (-8.6 to 5.6)	0.68	-2.9 (-9.9 to 4.1)	0.42	-2.5 (-9.3 to 4.4)	0.48
Pain VAS at activity	-3.2 (-13.0 to 6.6)	0.52	-3.4 (-13.5 to 6.6)	0.50	-2.2 (-11.7 to 7.2)	0.64
Constant-Murley score	-1.5 (-8.2 to 5.2)	0.66	-2.2 (-9.0 to 4.5)	0.52	-2.9 (-9.3 to 3.5)	0.37
SST	0.0 (-0.9 to 0.9)	0.97	-0.1 (-1.0 to 0.8)	0.83	-0.1 (-1.0 to 0.7)	0.77
Satisfaction to the treatment (VAS 0-100)	4.7 (-2.5 to 12.0)	0.20	5.7 (-1.5 to 13.0)	0.12	5.6 (-1.4 to 12.6)	0.12
15D	-0.003 (-0.023 to 0.016)	0.75	-0.003 (-0.023 to 0.018)	0.81	-0.005 (-0.024 to 0.014)	0.62
SF-36 score Physical health<br>Mental health	-0.8 (-5.6 to 3.9)<br>-1.8 (-6.3 to 2.7)	0.73<br>0.44	-1.4 (-6.1 to 3.4)<br>-2.4 (-7.1 to 2.2)	0.57<br>0.30	-2.7 (-7.3 to 1.9)<br>-2.3 (-6.7 to 2.1)	0.25<br>0.30

* ITT, the intention to treat population (ASD: n = 56, Placebo surgery: n = 55).

† The per protocol population is the subset of the intention to treat population who received the treatment they were randomised to and who did not receive any other treatment, i.e. the patients with a treatment conversion have been excluded (ASD: n=56, Placebo surgery: n=47).

‡ The as treated population is defined according to the treatment the participants received, i.e. the participants who originally received placebo surgery or ET, but due to persistent symptoms requested unblinding and subsequently received ASD, have been included in the ASD population (ASD: n=79, Placebo surgery: n=47).

Table S6 Sensitivity analyses of the primary and secondary outcomes concerning the secondary comparison (ASD vs. ET)
The table shows the between-group differences at the 10-year follow-up. The estimated effect indicates the mean difference, ASD minus ET. The analyses were carried out using a mixed model repeated measures (MMRM) regression with adjustment for baseline imbalance.

**Variable**	**FAS Estimated effect (95% CI)***	**P value**	**Per protocol Estimated effect (95% CI)†**	**P value**	**As treated Estimated effect (95% CI)‡**	**P value**
Pain VAS at rest	-4.0 (-11.0 to 3.0)	0.26	-3.6 (-10.6 to 3.5)	0.32	-3.2 (-10.1 to 3.7)	0.36
Pain VAS on arm activity	-9.4 (-19.0 to 0.3)	0.06	-10.4 (-20.6 to -0.2)	**0.04**	-9.2 (-18.8 to 0.4)	0.06
Constant-Murley score	4.1 (-2.5 to 10.8)	0.22	4.7 (-2.2 to 11.5)	0.18	4.1 (-2.4 to 10.7)	0.22
SST	0.3 (-0.6 to 1.2)	0.57	0.4 (-0.5 to 1.4)	0.38	0.4 (-0.5 to 1.3)	0.38
Satisfaction to treatment (0-100 VAS)	1.9 (-5.3 to 9.0)	0.61	2.6 (-4.8 to 10.0)	0.49	2.5 (-4.6 to 9.6)	0.49
15D	-0.006 (-0.025 to 0.013)	0.55	-0.014 (-0.034 to 0.07)	0.19	-0.016 (-0.036 to 0.003)	0.10
SF-36 score Physical health	0.0 (-4.7 to 4.7)	0.99	-2.2 (-7.1 to 2.8)	0.39	-3.4 (-8.2 to 1.3)	0.16
Mental health	-2.8 (-7.3 to 1.6)	0.21	-3.2 (-8.0 to 1.6)	0.19	-3.0 (-7.5 to 1.5)	0.19

* FAS, the full analysis set population (ASD: n = 56, ET: n = 58).

† The per protocol population is the subset of the full analysis set population who received the treatment they were randomised to and who did not receive any other treatment, i.e. the patients with a treatment conversion have been excluded (ASD: n=56, ET: n=43).

‡ The as treated population is defined according to the treatment the participants received, i.e., the participants who originally received placebo surgery or ET, but due to persistent symptoms requested unblinding and subsequently received ASD, have been included in the ASD population (ASD: n=79, ET: n=43).

Table S7 Unblindings, treatment conversions, and reoperations

Study group	Unblinding or decision on treatment conversion. Time from randomisation (months)	Treatment converted to*	Reoperations	Time from randomisation to reoperation (months)
ET	2	ASD		
ET	5	ASD	MUA and ADCR	10
ET	6	ASD and MUA		
ET	6	ASD	RC repair	23
ET	7	ASD		
ET	7	ASD		
ET	7	ASD	ASD and LHBT	23
ET	7	ASD		
ET	7	ASD		
ET	8	ASD		
ET	8	ASD		
ET	9	ASD	ASD and ADCR	57
ET	11	ASD		
ET	11	ASD		
ET	12	ASD		
ET	15	ADCR		
ET	21	ACR and ASD		
ET	34	ASD, LHBT and RC repair		
ET	55	ASD		
ASD	3			
ASD	8			
ASD	8	MUA		
ASD	13			
ASD	14			
ASD	18	ADCR	ASD and ADCR	51
ASD	67	ASD, LHBT and ADCR	ASD, ADCR	77
ASD	72	ASD and RC repair		
Placebo surgery	1	ASD		
Placebo surgery	5	ASD		
Placebo surgery	6			
Placebo surgery	9	ASD		
Placebo surgery	9	ASD		
Placebo surgery	9	ASD		
Placebo surgery	11	ASD		
Placebo surgery	12	ASD		
Placebo surgery	19	ASD and RC repair		
Placebo surgery	25			
Placebo surgery	26	ASD and DPC		
Placebo surgery	52	RC repair	Debridement	72
Placebo surgery	55	RC repair		
Placebo surgery	No unblinding	RC repair		
Placebo surgery	80	ASD		
Placebo surgery	83	ASD and RC repair		

Abbreviations: ASD = Arthroscopic Subacromial Decompression; ET = Exercise therapy ; ACR = Arthroscopic Capsular Release; MUA = Manipulation Under Anesthesia; ADCR = Arthroscopic Distal Clavicle Resection; LHBT = Long Head of the Biceps Tendon; RC repair = Rotator Cuff repair; DPC = Drainage of a Paralabral Cyst.

*Treatment conversions (surgeries) were carried out within 6 months of unblinding (mean: 8 weeks, range 1-24 weeks).

Table S8 Frequency of missing data at baseline, 3, 6, 12, 24 months, 5 and 10 years						
**Study group**	**Timepoint**	**Pain VAS at rest**	**Pain VAS on arm activity**	**Constant-Murley score**	**SST**	**15D**
ASD (n=59)	Baseline	0	0	0	0	2
	3 months	5	7	N/A	N/A	2
	6 months	0	0	0	0	3
	12 months	4	4	N/A	N/A	2
	24 months	0	0	1	1	1
	5 years	5	5	5	7	6
	10 years	11	11	14	11	3
Placebo surgery (n=63)	Baseline	0	0	0	0	3
	3 months	8	8	N/A	N/A	6
	6 months	2	2	2	2	5
	12 months	7	7	N/A	N/A	8
	24 months	4	4	4	4	5
	5 years	9	9	9	10	12
	10 years	12	11	14	11	3
ET (n=71)	Baseline	1	1	1	1	2
	3 months	9	9	N/A	N/A	6
	6 months	3	3	3	3	5
	12 months	6	7	N/A	N/A	7
	24 months	3	3	6	5	3
	5 years	9	9	11	9	12
	10 years	16	16	18	16	15

Abbreviations: ASD = arthroscopic subacromial decompression; ET = exercise therapy; VAS = visual analogue scale; SST = simple shoulder test. The n values denote the number of participants allocated to each treatment group. The frequencies include both missing data and withdrawn subjects.

Table S9 Key Concerns Regarding Prior Publications of the FIMPACT Trial		
**Concern**	**Rationale for the assertion**	**Authors' response**
Diagnostic arthroscopy is not a true placebo comparator	Because joint lavage allegedly has a therapeutic effect[1-4], diagnostic arthroscopy cannot be considered a true placebo comparator.	Previous studies have shown that tidal irrigation and arthroscopic lavage have both failed to provide a benefit over placebo irrigation or skin incisions[5 6].
Sample size: underpowered study?	The final sample size was below pre-specified target, raising concerns that the study may lack statistical power to detect meaningful differences.	Had we simply used the more conventional 80% in estimating sample size *a priori*—instead of the more stringent 90% we used—and maintained all other criteria constant, we would have met the recruitment target with the numbers we recruited.<br><br>Moreover, an increase in sample size simply reduces the width of confidence interval (CI) but does not alter the effect estimates. The absence of between-group differences, combined with point estimates excluding clinically meaningful effects, suggests that our findings are not based on absence of evidence, as in an underpowered study, but rather on evidence of absence of a clinically significant treatment benefit.
Validity of the minimal clinically important difference (MCID) threshold	The threshold for clinically important improvement (15 VAS points) was chosen without prior validation for this patient population.	At trial design, no validated MCID existed for shoulder impingement syndrome, and accordingly, our MCID estimates were based on a comprehensive literature review.<br><br>More recent studies confirmed an MCID of 14 VAS points in rotator cuff disease[7] and even higher for pain in arm activity (20 points) in patients with subacromial pain syndrome (FIMPACT trial sample[8]), validating the MCID thresholds used in the FIMPACT trial.

Partial subacromial bursectomy performed in some participants	Some participants underwent partial bursectomy for better visualization of the rotator cuff.<br><br>Given that previous studies have suggested that bursectomy alone (complete resection of the subacromial bursa) provides similar outcomes to subacromial decompression (bursectomy accompanied by resection of acromial bone) in patients with shoulder impingement syndrome[9-11], it was asserted that partial bursectomy performed in some patients skewed our results.	Bursectomy was performed in 30% (18/63) of diagnostic arthroscopy patients and was minimal in all but three cases. To assess the possible effect of this bursal tissue resection on our findings, we carried out a pre-specified post hoc analysis, which did not show any statistically significant differences in the primary outcomes between patients who had resection carried out and those who did not. If anything, the observed marginal differences favoured no resection.
Prognostic imbalance due to exclusion of participants during diagnostic arthroscopy	Approximately 4% (6/134) of surgical participants were excluded due to rotator cuff tears discovered at diagnostic arthroscopy, potentially affecting group comparability (ET vs. ASD and Placebo surgery).	The ASD (or Placebo surgery) and exercise therapy (ET) groups were not fully comparable due to different interventions. However, this exclusion does not impact the primary ASD vs. Placebo surgery comparison.
Misclassification of frozen shoulder cases	Some participants diagnosed with frozen shoulder as a treatment complication may have initially had undiagnosed frozen shoulder instead of impingement syndrome.	The number of frozen shoulder cases was low across all groups (ET: 2, ASD: 3, Placebo surgery: 1), making misclassification unlikely to substantially impact results.
Pain at rest not a valid primary outcome measure	Pain at rest has low responsiveness, and no minimum pain threshold was set for study inclusion, meaning some participants had little room for improvement.	Even in the other primary outcome (pain on activity) and all secondary outcomes, ASD showed no clinically meaningful benefit, reinforcing our conclusion.
Group differences below the MCID threshold may still mask important responder differences	While mean differences between groups may not exceed MCID, there could still be differences in the proportion of patients achieving clinically meaningful improvement.	A responder analysis was conducted, comparing the proportion of patients satisfied with treatment outcomes. This analysis showed no significant difference between groups.
Higher crossover rates suggest surgery may be superior	Some argue that the higher frequency of crossovers (patients switching from conservative to surgical treatment) implies that surgery is more effective.	Crossover decisions were made after unblinding, whereas decisions to unblind were made blinded to treatment allocation. A more unbiased measure of symptom severity is the rate of unblindings, which did not differ significantly between ASD and placebo surgery groups.

1. Sihvonen R, Paavola M, Malmivaara A, et al. Arthroscopic partial meniscectomy versus sham surgery for a degenerative meniscal tear. N Engl J Med 2013;369(26):2515-24. doi: 10.1056/NEJMoa1305189 [published Online First: 2013/12/27]
2. Schroder CP, Skare O, Reikeras O, et al. Sham surgery versus labral repair or biceps tenodesis for type II SLAP lesions of the shoulder: a three-armed randomised clinical trial. Br J Sports Med 2017 doi: 10.1136/bjsports-2016-097098
3. Lattermann C, Gomoll AH, Cole BJ. Arthroscopic partial meniscectomy for degenerative meniscal tear. N Engl J Med 2014;370(13):1260. doi: 10.1056/NEJMc1401128#SA3
4. Brophy R. Arthroscopic partial meniscectomy was not better than sham surgery for medial meniscal tear. J Bone Joint Surg Am 2014;96(16):1396. doi: 10.2106/JBJS.9616.ebo521
5. Moseley JB, O'Malley K, Petersen NJ, et al. A controlled trial of arthroscopic surgery for osteoarthritis of the knee. N Engl J Med 2002;347(2):81-8. doi: 10.1056/NEJMoa013259
6. Bradley JD, Heilman DK, Katz BP, et al. Tidal irrigation as treatment for knee osteoarthritis: a sham-controlled, randomized, double-blinded evaluation. Arthritis Rheum 2002;46(1):100-8. doi: 10.1002/1529-0131(200201)46:1<100::aid-art10037>3.0.co;2-v
7. Tashjian RZ, Deloach J, Porucznik CA, et al. Minimal clinically important differences (MCID) and patient acceptable symptomatic state (PASS) for visual analog scales (VAS) measuring pain in patients treated for rotator cuff disease. J Shoulder Elbow Surg 2009;18(6):927-32. doi: 10.1016/j.jse.2009.03.021 [published Online First: 2009/06/19]
8. Kanto K, Lahdeoja T, Paavola M, et al. Minimal important difference and patient acceptable symptom state for pain, Constant-Murley score and Simple Shoulder Test in patients with subacromial pain syndrome. BMC Med Res Methodol 2021;21(1):45. doi: 10.1186/s12874-021-01241-w [published Online First: 20210306]
9. Henkus HE, de Witte PB, Nelissen RG, et al. Bursectomy compared with acromioplasty in the management of subacromial impingement syndrome: a prospective randomised study. J Bone Joint Surg Br 2009;91(4):504-10. doi: 10.1302/0301-620X.91B4.21442
10. Donigan JA, Wolf BR. Arthroscopic subacromial decompression: acromioplasty versus bursectomy alone--does it really matter? A systematic review. Iowa Orthop J 2011;31:121-6. [published Online First: 2011/11/19]
11. Clement ND, Watts AC, Phillips C, et al. Short-Term Outcome After Arthroscopic Bursectomy Debridement of Rotator Cuff Calcific Tendonopathy With and Without Subacromial Decompression: A Prospective Randomized Controlled Trial. Arthroscopy 2015;31(9):1680-7. doi: 10.1016/j.arthro.2015.05.015

Exercise Therapy (ET) Protocol

**Phase I (weeks 0-3)**	
**Goals: Decrease pain, restore A/PROM, relieve pain and inflammation, re-establish muscular balance, and improve posture; Inform the patient**	
**Passive exercises:**<br>PROM<br>Joint mobilisation/posterior capsule stretching<br>Soft tissue mobilisation	**Active exercises:**<br>AAROM<br>Submaximal isometric rotator cuff exercises<br>Thoracic spine mobilisation<br>Scapular retraction/protraction
**Phase II (weeks 4-5)**	
**Goals: Re-establish full and pain free AROM, restore rotator cuff strength, restore normal scapulothoracic motion**	
**Passive exercises:**<br>Joint mobilisation/posterior capsule stretching<br>Soft tissue mobilisation	**Active exercises:**<br>AROM<br>Maximal isometric rotator cuff exercises<br>Thoracic spine mobilisation<br>Scapulothoracic motion
**Phase III (weeks 6-8)**	
**Goals: Restore muscle strength and endurance, re-establish full and pain free AROM. Restore normal scapulothoracic motion**	
**Passive exercises:**<br>Joint mobilisation	**Active exercises:**<br>Dynamic rotator cuff exercises<br>Scapulothoracic motion
**Phase IV (weeks 9-12)**	
**Goals: Enhance muscle strength and endurance, re-educate neuromuscular control of rotator cuff muscles**	
**Passive exercises:**<br>Continued stretching program	**Active exercises:**<br>Continued dynamic rotator cuff program

Abbreviations: PROM, Passive range of motion; AAROM, Active-assisted range of motion; AROM: Active range of motion

Exercise therapy (ET) group: home exercise program

Phase I (0-3 weeks)

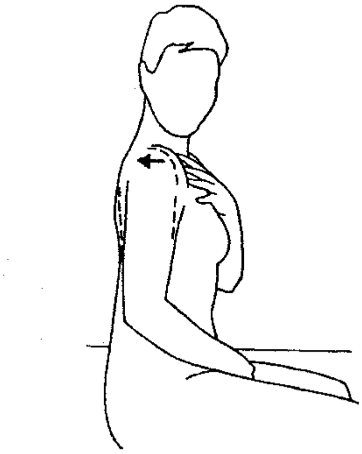

© PhysioTools Ltd

Sit. Place your hand on the front of the opposite shoulder. Feel your shoulder with your fingers making sure that the shoulder does not come forward. Move your shoulder gently 1 cm back and 2 cm up. Later on, exercise is done without palpation or visual confirmation of movement.

Repeat 3 x 15-25 times.

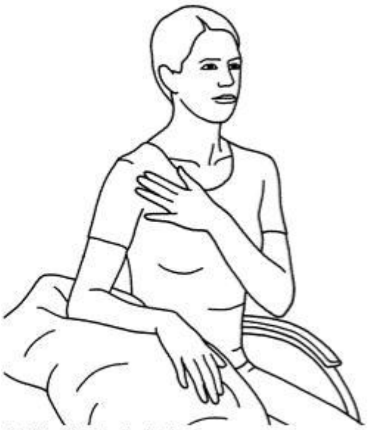

©PhysioTools Ltd

Sit. Place your hand on the front of the opposite shoulder. Feel your shoulder with your fingers. Move your shoulder gently 1 cm forward and 2 up from resting position. Later on, exercise is done without palpation or visual confirmation of movement.

Repeat 3 x 15-25 times.

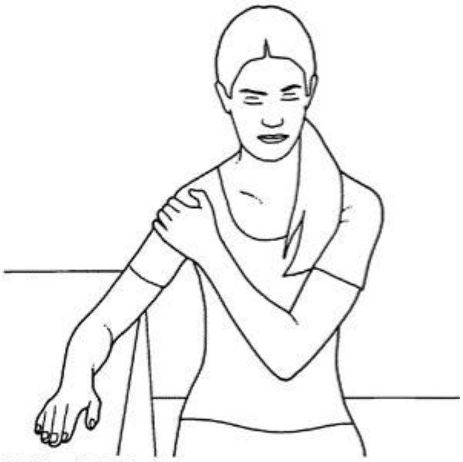

©PhysioTools Ltd

Sit on a chair with your arm supported on a table.
With your other hand push the top of your upper arm downwards.
Hold approx. 2 secs

Repeat 10 times.

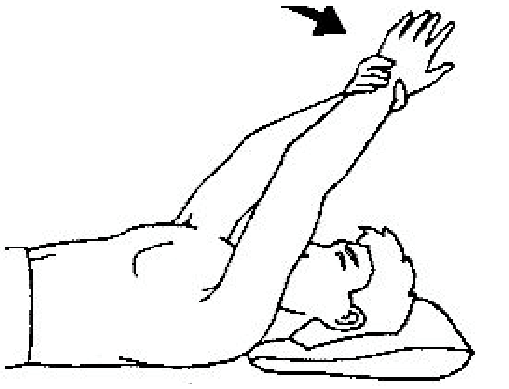

© PhysioTools Ltd

Lying on your back with elbows straight. Use one arm to lift the other arm up keeping it as close to the ear as possible.

Repeat 10 times.

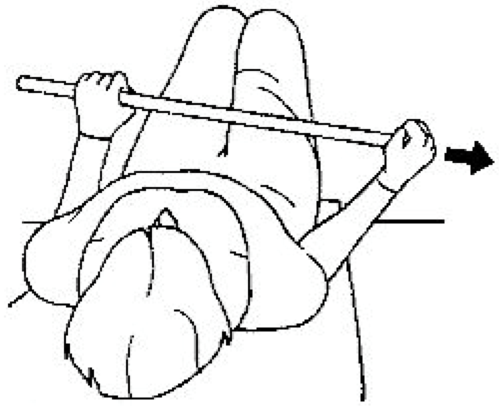

© PhysioTools Ltd

Lying on your back with elbows against your body and at a right angle. Hold a stick in your hands. Move the stick sideways thus pushing the arm to be exercised outwards.

Repeat 10 times.

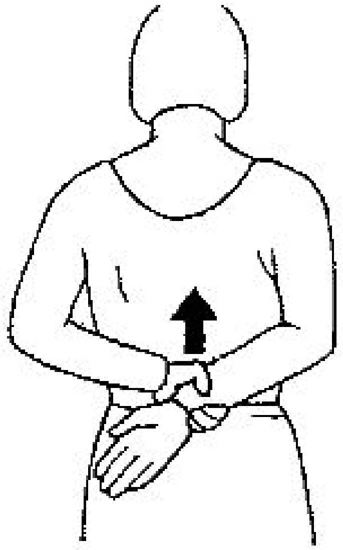

© PhysioTools Ltd

Stand with arms behind your back. Grasp the wrist of the arm you want to exercise. Slide your hands up the back.

Repeat 10 times.

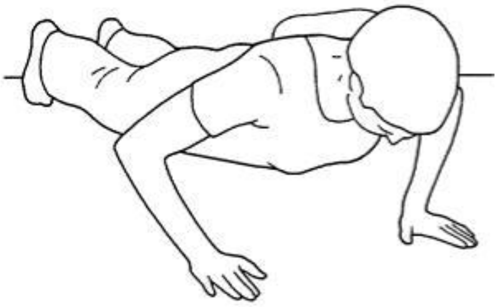

©PhysioTools Ltd

Lying face down with your hands on the floor at shoulder height. Do push-ups slowly and remember to straighten your elbows properly.

Repeat 3 x 15-25 times.

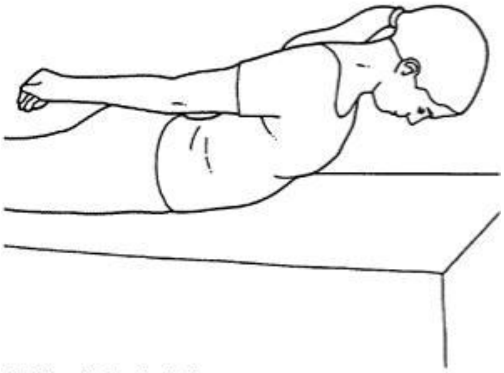

©PhysioTools Ltd

Lying face down, arms behind your back. Lift your upper trunk off the floor and pull your shoulder blades together. Look down at the floor while doing the exercise.

Repeat 3 x 15-25 times.

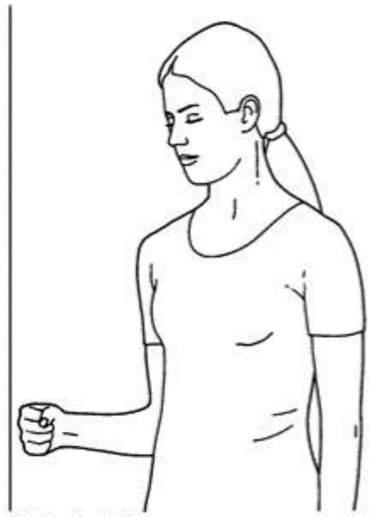

©PhysioTools Ltd

Stand sideways against a wall with your upper arm close to your side and elbow at a right angle. Push the forearm to the side against the wall. Hold approx. 5 secs.

Repeat 3 x 3 times.

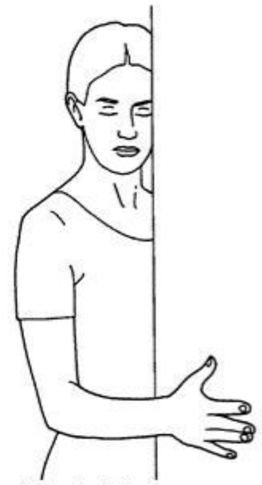

©PhysioTools Ltd

Stand in a doorway with you elbow close to your body and bent at a right angle. Place your hand against the wall. Push your hand inwards against the wall. Hold 5 secs. Relax.

Repeat 3 x 3 times.

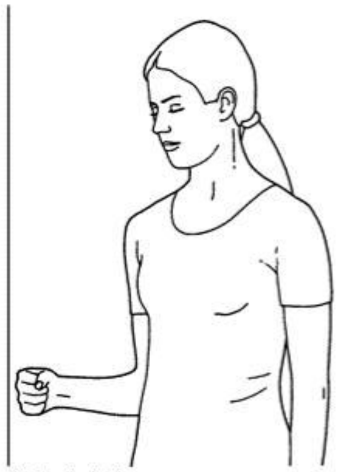

©PhysioTools Ltd

Stand with your upper arm close to your side, elbow at a right angle and the back of your hand against a wall. Push the back of your hand against the wall. Hold approx. 5secs.

Repeat 3 x 3times.

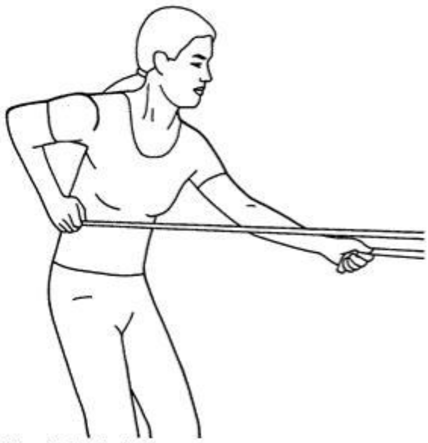

©PhysioTools Ltd

Stand facing a rubber exercise band with your knees and hips slightly bent. Pull the band alternately with the left and right hand keeping the pelvis still.

Repeat 3 x 15-25 times.

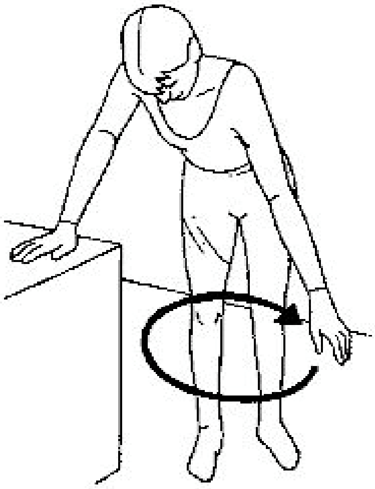

© PhysioTools Ltd

Stand leaning on a table with one hand. Let your other arm hang relaxed straight down. Swing your arm as if drawing a circle on the floor. Change direction.

Continue for 1-2 minutes

Phase II (4-5 weeks)

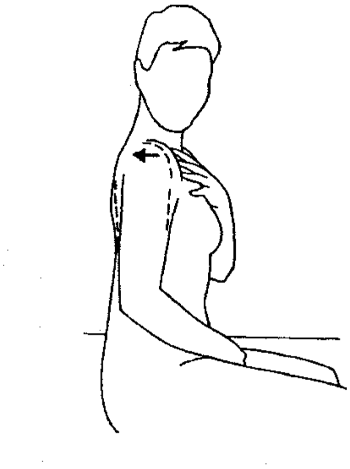

© PhysioTools Ltd

Sit. Place your hand on the front of the opposite shoulder. Feel your shoulder with your fingers making sure that the shoulder does not come forward. Move your shoulder gently 1 cm back and 2 cm up. Later on, exercise is done without palpation or visual confirmation of movement.

Repeat 3 x 15-25 times.

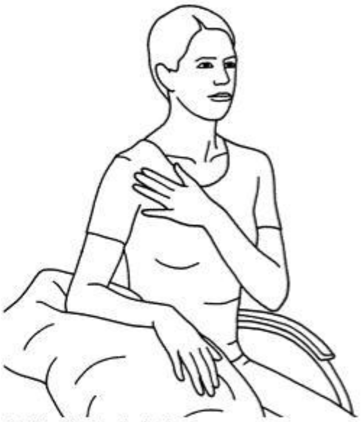

©PhysioTools Ltd

Sit. Place your hand on the front of the opposite shoulder. Feel your shoulder with your fingers. Move your shoulder gently 1 cm forward and 2 up from resting position. Later on, exercise is done without palpation or visual confirmation of movement.

Repeat 3 x 15-25 times.

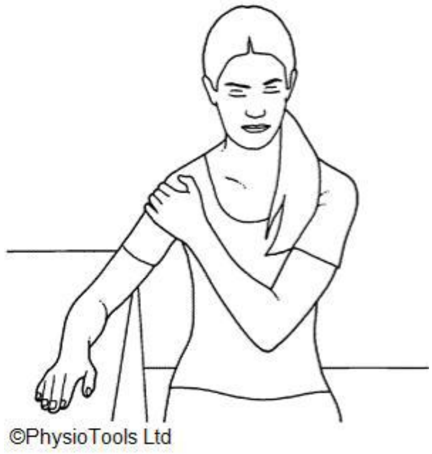

Sit on a chair with your arm supported on a table.
With your other hand push the top of your upper arm downwards. Hold approx. 2 secs.

Repeat 10 times.

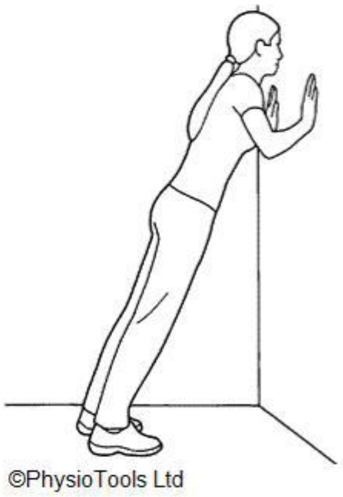

Stand facing a wall with your arms straight and hands on the wall. Do push-ups against the wall keeping your body in a straight line.

Repeat 3 x 15-25 times.

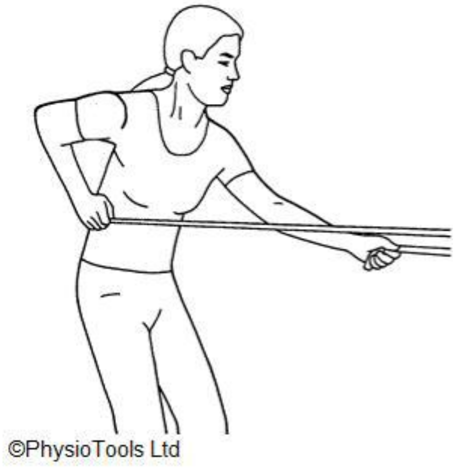

Stand facing a rubber exercise band with your knees and hips slightly bent.
Pull the band alternately with the left and right hand keeping the pelvis still.

Repeat 3 x 15-25 times.

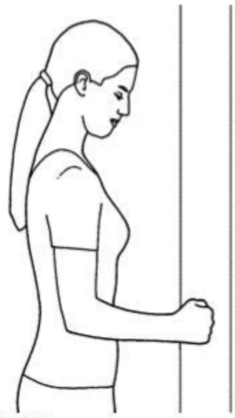

©PhysioTools Ltd

Stand facing a wall. Keep your upper arm close to the side with elbow at a right angle. Push your fist against the wall for 5 secs.

Repeat 3 x 3 times.

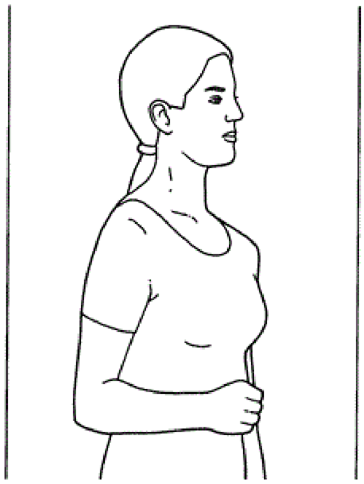

©PhysioTools Ltd

Stand with your back against the wall. Keep your upper arm close to the side and elbow at a right angle. Push the elbow back against the wall and hold for 5 secs.

Repeat 3 x 3 times.

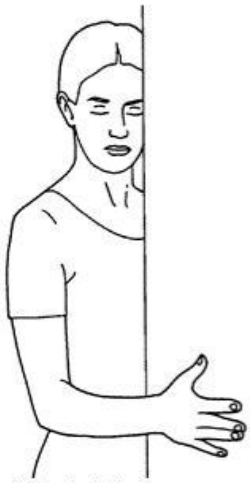

©PhysioTools Ltd

Stand in a doorway with you elbow close to your body and bent at a right angle. Place your hand against the wall. Push your hand inwards against the wall. Hold 5 secs.

Repeat 3 x 3 times.

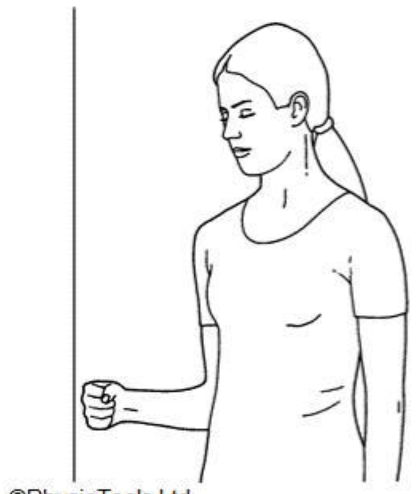

©PhysioTools Ltd

Stand with your upper arm close to your side, elbow at a right angle and the back of your hand against a wall. Push the back of your hand against the wall. Hold approx. 5 secs.

Repeat 3 x 3times.

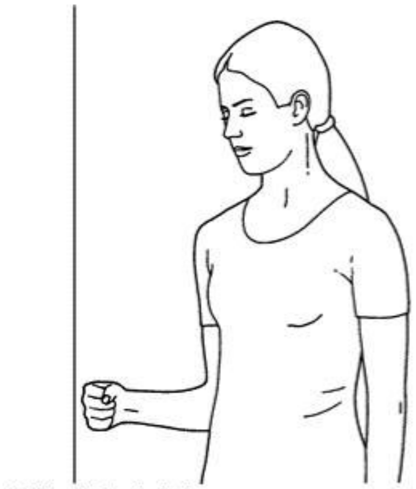

©PhysioTools Ltd

Stand sideways against a wall with your upper arm close to your side and elbow at a right angle. Push the forearm to the side against the wall. Hold approx. 5 secs.

Repeat 3 x 3 times.

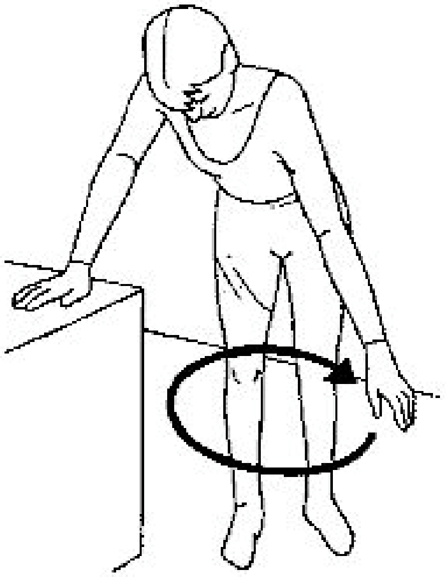

© PhysioTools Ltd

Stand leaning on a table with one hand. Let your other arm hang relaxed straight down. Swing your arm as if drawing a circle on the floor. Change direction.

Continue for 1-2 minutes

Phase III (6-8 weeks)

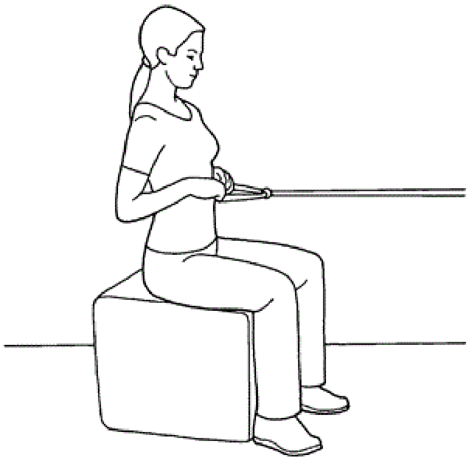

©PhysioTools Ltd

Sit or stand holding on to a rubber exercise band with both hands. Pull the band with both arms pushing the shoulder blades together.

Repeat 3 x 15-25 times.

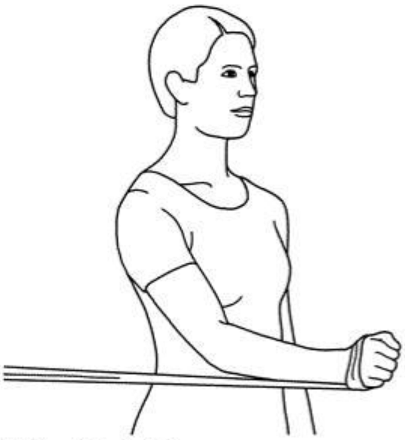

©PhysioTools Ltd

Stand straight holding an exercise band. Move your arm forward, pulling the band. Slowly return.

Repeat 3 x 15-25 times.

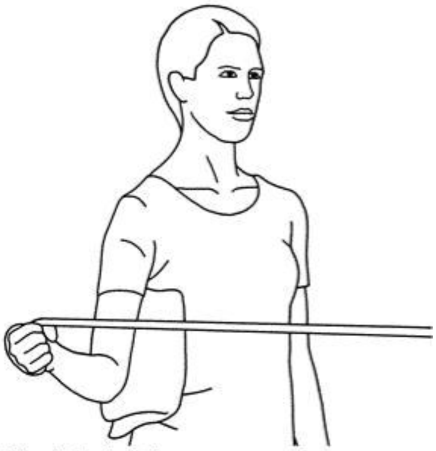

©PhysioTools Ltd

Stand or sit. Place a rolled towel between your upper arm and side. Your upper arm should be slightly forward and your elbow at a right angle. Hold an exercise band. Pull the band taking your forearm out 45 degrees. Hold for 5 secs.

Repeat 3 x 15-25 times.

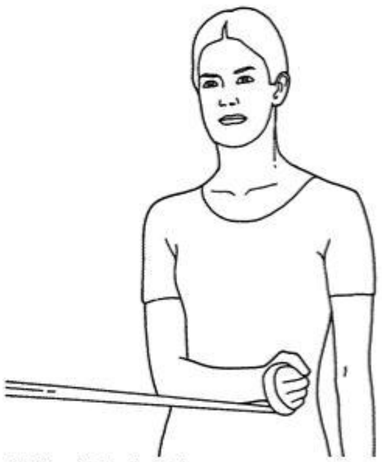

©PhysioTools Ltd

Stand keeping your upper arm close to the side and elbow at a right angle. Hold a rubber exercise band. Pull the band toward your stomach and hold for 5 secs.

Repeat 3 x 15-25 times.

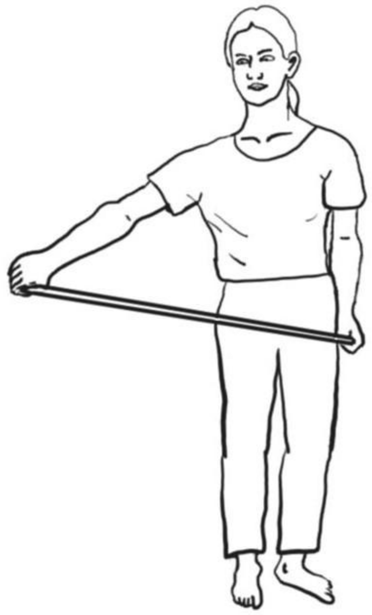

Hold an exercise band with both hands. Pull the band outwards to about 45-60 degrees with back of your hand leading the movement. Hold for 5 secs.

Repeat 3 x 15-25 times

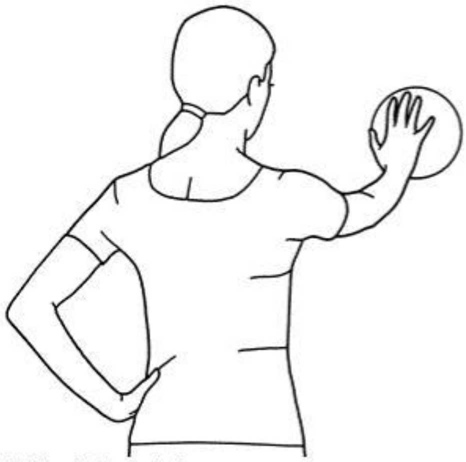

©PhysioTools Ltd

Stand with one foot forward. Keep your back straight. Place the palm of your hand against a wall using a ball or a piece of cloth. Lean your body weight onto your hand. Control your shoulder position and move hand in small horisontal motions.

Repeat 3 x 15-25 times.

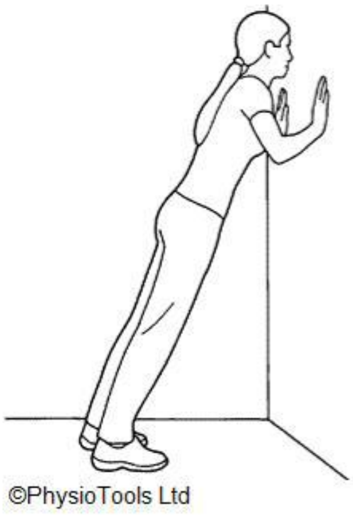

Stand facing a wall with your arms straight and hands on the wall. Do push-ups against the wall keeping your body in a straight line.

Repeat 3 x 15-25 times.

Phase IV (9-12 weeks)

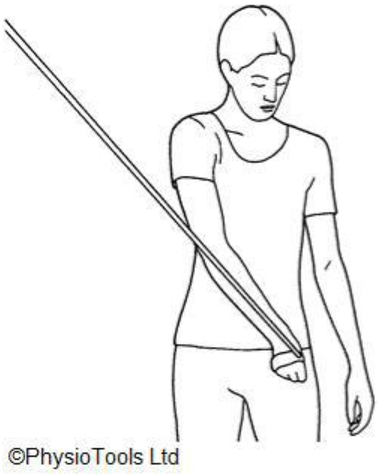

Stand with your arm up and out to the side. Hold a rubber exercise band. Pull the band down and across your body letting your thumb lead the movement.

Repeat 3 x 15-25 times.

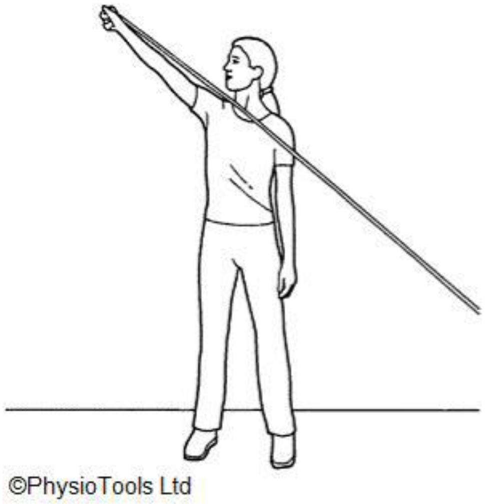

Stand or sit with the hand of the arm to be exercised on your opposite hip. Hold on to a rubber exercise band. Pull the band up towards the opposite side.

Repeat 3 x 15-25 times.

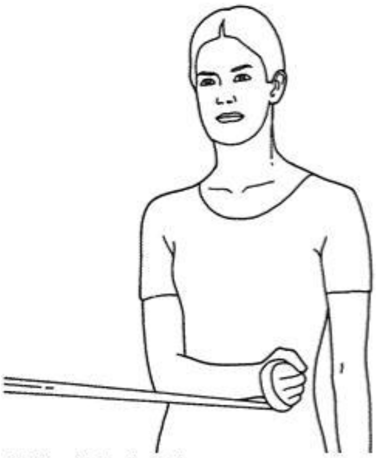

©PhysioTools Ltd

Stand keeping your upper arm close to the side and elbow at a right angle. Hold a rubber exercise band. Pull the band toward your stomach.

Repeat 3 x 15-25 times.

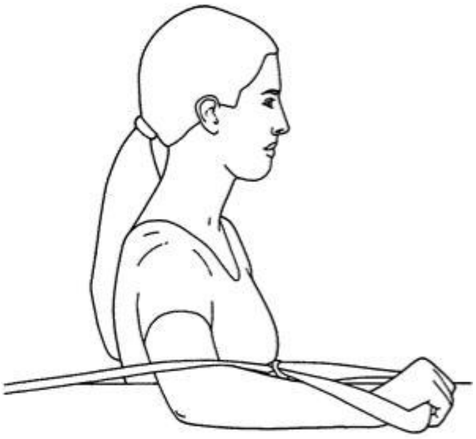

©PhysioTools Ltd

Alternatively,
Sit on a chair with your arm lifted to the side and your elbow at a right angle supported on a table. Hold on to a rubber exercise band which is fastened behind you. Pull the band keeping elbow bent and resting on the table.

Repeat 3 x 15-25 times.

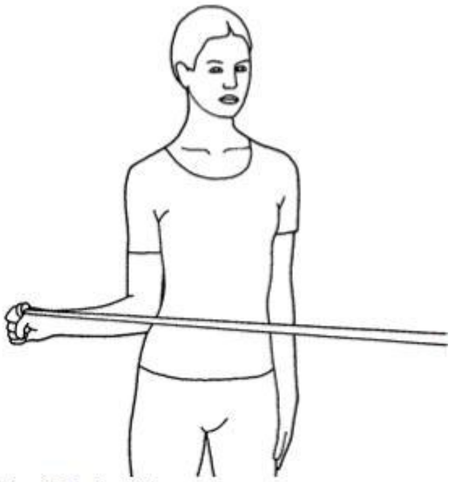

©PhysioTools Ltd

Stand keeping your upper arm close to the side and elbow at a right angle. Hold a rubber exercise band. Pull the band by turning your forearm outwards.

Repeat 3 x 15-25 times.

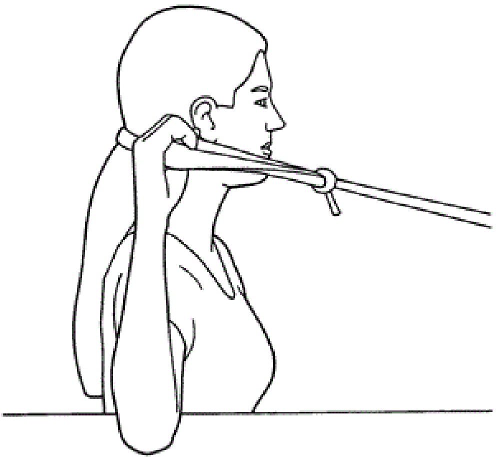

©PhysioTools Ltd

Alternatively,
Sit on a chair with your arm lifted out to the side and elbow at a right angle supported on a table. Hold on to a rubber exercise band which is in front on you. Pull the band keeping your elbow bent and resting on the table.

Repeat 3 x 15-25 times.

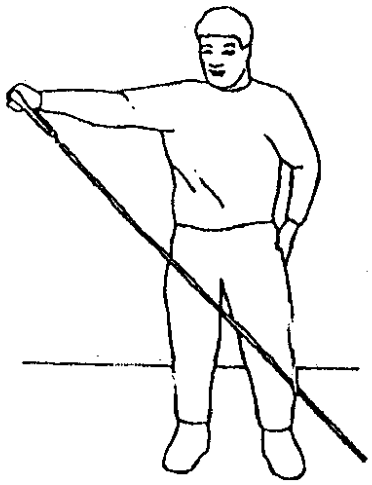

© PhysioTools Ltd

Stand or sit with the hand of the arm to be exercised at your side. Hold on to a rubber exercise band attached at floor level. Pull the band up upwards up to 90 degrees.

Repeat 3 x 15-25 times.

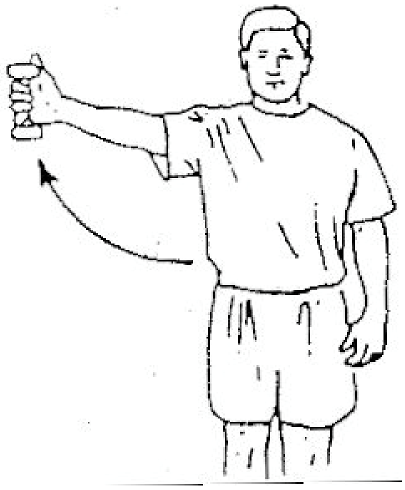

© PhysioTools Ltd

Stand or sit with the hand of the arm to be exercised at your side. Lift hand in thump up position up to a 90° of abduction.

Repeat 3 x 15-25 times.

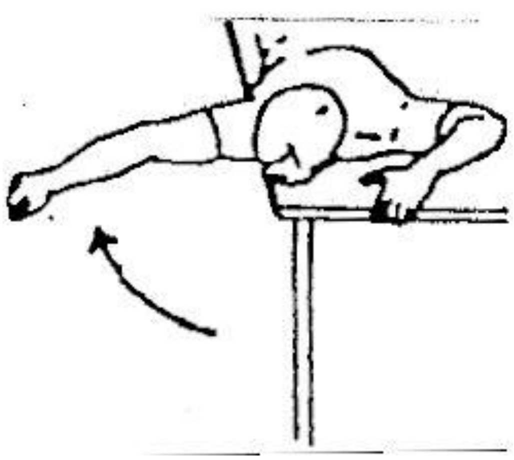

© PhysioTools Ltd

Lying face down with your arms out to the side. Lift your arm toward the ceiling with back of your hand leading the movement.

Repeat 3 x 15-25 times.

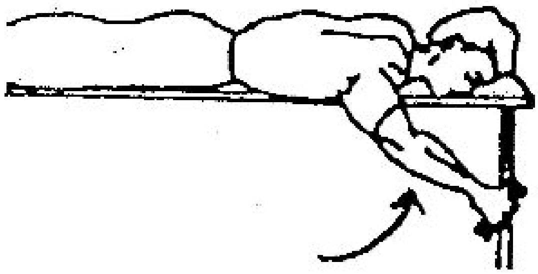

© PhysioTools Ltd

Lying face down with your arms out to the side. Lift your arm forwards up to 100° of flexion with your thumbs leading the movement.

Repeat 3 x 15-25 times.

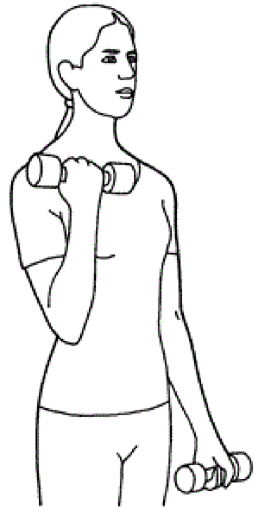

©PhysioTools Ltd

Stand with arms hanging down. Hold a hand weights and turn your palms forward. Bend alternate elbows briskly.

Repeat 3 x 15-25 times.

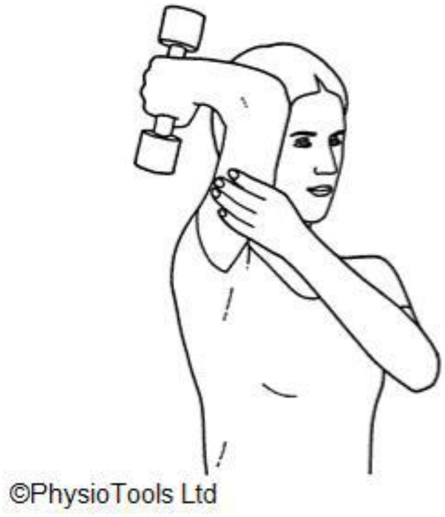

Sit or stand holding a kg hand weight. Bring the arm to be exercised up with the elbow pointing to the ceiling. Support the elbow with the other hand. Straighten the arm holding the weight.

Repeat 3 x 15-25 times.

Statistical Analysis Plan for the FIMPACT Trial at the 10-year Follow-Up

Taimela S, Kanto K, Czuba T, Paavola M, Järvinen TLN for FIMPACT Investigators *Version: 8 July 2024*

Study question	Comparison	Outcomes	Analysis
Efficacy: Is ASD superior to Placebo surgery?	ASD vs. Placebo surgery	Primary and secondary outcomes as reported at 2 and 5 years	RMMM or log reg when applicable (intention-to-treat)
Pragmatic: Is ASD superior to ET?	ASD vs. ET		
Exploratory: Does ASD protect from rotator cuff tears?	ASD vs. no ASD	Rotator cuff tear (RCT) in magnetic resonance imaging (MRI)	Comparison of proportion of RCTs among those who had and had not received ASD during the follow-up.
Exploratory: Is ASD superior to no ASD?	ASD vs. no ASD	Primary and secondary outcomes as reported at 2 and 5 years	RMMM or log reg when applicable

Outcomes:

Primary: Shoulder pain at rest and shoulder pain on arm activity, assessed with Visual Analogue Scale (VAS) ranging from 0 (no pain) to 100 (extreme pain).

Secondary: Constant-Murley score, Simple Shoulder Test, SF-36(R) Health Survey, 15D HRQoL instrument, and patients' global assessment of satisfaction to the treatment with 0-100 VAS scale, all continuous variables. Responder analysis: satisfaction with the treatment outcome on a 5-item scale, where very satisfied or satisfied were categorised as 'Responders'. Return to hobbies.
Full thickness rotator cuff tear in MRI.

Introduction

The FIMPACT trial is a superiority trial that was conducted at three orthopaedic clinics in Finland from 1 February 2005 to 20 September 2023. The trial was primarily designed to ascertain whether arthroscopic subacromial decompression (ASD) was superior to diagnostic arthroscopy (Placebo surgery) for pain reduction after the procedure (the primary confirmatory comparison). We also included a pragmatic comparison of the relative benefits of ASD vs exercise therapy (the secondary exploratory comparison). The participants in the two surgical groups and the people who collected the data were unaware of the study group assignments. The trial was conducted in accordance with the Declaration of Helsinki.

We enrolled patients aged 35–65 years who had subacromial pain for more than 3 months that was unresponsive to conventional conservative treatment, and clinical findings consistent with shoulder impingement syndrome. All patients gave written informed consent. On entering the study, they were unequivocally informed that they might undergo Placebo surgery and that they would be allowed to consider crossing over to ASD if they did not have

adequate relief of symptoms, preferably no sooner than 6 months after randomisation. Details of the trial design and conduct, and results at 2-year and 5-year follow-up have been published (REFS).

At the primary two-year time point, in the primary intention to treat analysis (ASD versus diagnostic arthroscopy), no clinically relevant between group differences were seen in the two primary outcomes at 24 months (mean change for ASD 36.0 at rest and 55.4 on activity; for diagnostic arthroscopy 31.4 at rest and 47.5 on activity). The observed mean difference between groups (ASD minus diagnostic arthroscopy) in pain VAS were −4.6 (95% confidence interval −11.3 to 2.1) points (P=0.18) at rest and −9.0 (−18.1 to 0.2) points (P=0.054) on arm activity. No between group differences were seen between the ASD and diagnostic arthroscopy groups in the secondary outcomes or adverse events. In the secondary comparison (ASD versus exercise therapy), statistically significant differences were found in favour of ASD in the two primary outcomes at 24 months in both VAS at rest (−7.5, −14.0 to −1.0, points; P=0.023) and VAS on arm activity (−12.0, −20.9 to −3.2, points; P=0.008), but the mean differences between groups did not exceed the prespecified minimal clinically important difference. Of note, this ASD versus exercise therapy comparison is not only confounded by lack of blinding but also likely to be biased in favour of ASD owing to the selective removal of patients with likely poor outcome from the ASD group, without comparable exclusions from the exercise therapy group.

At five years after the interventions, in the primary intention to treat analysis (ASD vs diagnostic arthroscopy), there were no between-group differences that exceeded the MID for the primary outcomes at 5 years: the mean difference between groups (ASD minus diagnostic arthroscopy) in pain VAS were −2.0 (95% CI −8.5 to 4.6; p=0.56) at rest and −8.0 (−17.3 to 1.3; p=0.093) on arm activity. There were no between-group differences in the secondary outcomes or adverse events that exceeded the MID. In the secondary comparison (ASD vs exercise therapy), the mean differences between groups (ASD minus exercise therapy) in pain VAS were 1.0 (−5.6 to 7.6; p=0.77) at rest and −3.9 (−12.8 to 5.1; p=0.40) on arm activity. There were no significant between-group differences for the secondary outcomes or adverse events.

In summary, ASD provided no clinically relevant benefit over diagnostic arthroscopy (or exercise therapy) at 2 or 5 years for patients with shoulder impingement syndrome.

Our study hypothesis is that ASD will not provide clinically relevant benefit over diagnostic arthroscopy or exercise therapy at 10 years either.

Sample size calculation

The study was originally powered to detect a difference of at least the MID (15 points) in the two primary outcomes (scale 0 to 100) between the ASD and diagnostic arthroscopy groups. For the study to have 90% power to show a minimal clinically important advantage of ASD over diagnostic arthroscopy, under the assumption of a two-sided type 1 error rate of 5%, we planned to recruit 70 participants per group.

Study flow at the 10-year follow-up

The number of subjects with some data available at the 10-year follow-up are 57 in the ET group, 55 in the Placebo surgery group, and 56 in the ASD group.

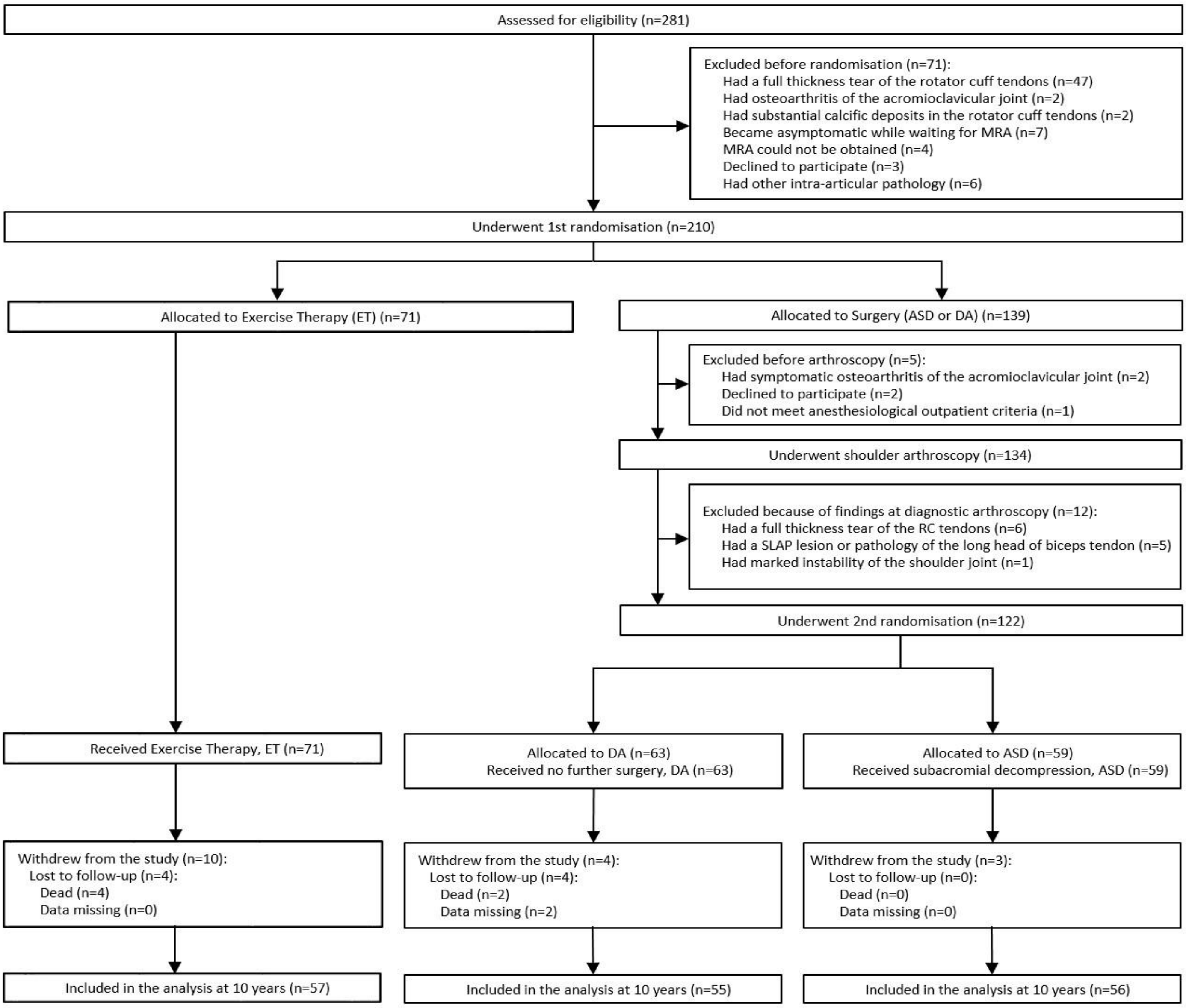
Assessed for eligibility (n=281)
Excluded before randomisation (n=71):
Had a full thickness tear of the rotator cuff tendons (n=47)
Had osteoarthritis of the acromioclavicular joint (n=2)
Had substantial calcific deposits in the rotator cuff tendons (n=2)
Became asymptomatic while waiting for MRA (n=7)
MRA could not be obtained (n=4)
Declined to participate (n=3)
Had other intra-articular pathology (n=6)
Underwent 1st randomisation (n=210)
Allocated to Exercise Therapy (ET) (n=71)
Allocated to Surgery (ASD or DA) (n=139)
Excluded before arthroscopy (n=5):
Had symptomatic osteoarthritis of the acromioclavicular joint (n=2)
Declined to participate (n=2)
Did not meet anesthesiological outpatient criteria (n=1)
Underwent shoulder arthroscopy (n=134)
Excluded because of findings at diagnostic arthroscopy (n=12):
Had a full thickness tear of the RC tendons (n=6)
Had a SLAP lesion or pathology of the long head of biceps tendon (n=5)
Had marked instability of the shoulder joint (n=1)
Underwent 2nd randomisation (n=122)
Received Exercise Therapy, ET (n=71)
Allocated to DA (n=63)
Received no further surgery, DA (n=63)
Allocated to ASD (n=59)
Received subacromial decompression, ASD (n=59)
Withdrew from the study (n=10):
Lost to follow-up (n=4):
Dead (n=4)
Data missing (n=0)
Withdrew from the study (n=4):
Lost to follow-up (n=4):
Dead (n=2)
Data missing (n=2)
Withdrew from the study (n=3):
Lost to follow-up (n=0):
Dead (n=0)
Data missing (n=0)
Included in the analysis at 10 years (n=57)
Included in the analysis at 10 years (n=55)
Included in the analysis at 10 years (n=56)

Outcome measures at the 10-year follow-up

Given that the pathognomonic clinical sign of shoulder impingement syndrome is subacromial shoulder pain, especially at night and while lifting the arm, our two primary outcome measures are shoulder pain at rest and shoulder pain on arm activity, according to the original study plan. We used a Visual Analogue Scale (VAS) ranging from 0 (no pain) to 100 (extreme pain) to measure the outcomes. We considered 15 points as the minimal important difference (MID) (ref).

The secondary outcomes are two shoulder function assessment instruments, the Constant-Murley score (CM) and the simple shoulder test (SST), and two health-related quality of life instruments, the SF-36(R) Health Survey (ref) and the 15D.(ref) MID was set at 17 points as for the CM (ref) and two points for SST (ref). Patients' global assessment of satisfaction to the treatment was elicited with this question: 'Are you satisfied with the treatment you have received?' We used a VAS scale ranging from 0 (completely disappointed) to 100 (very satisfied). Patients' satisfaction with the treatment outcome was elicited with a question: 'How satisfied are you with the outcome of your treatment?' on a 5-item scale. Patients who reported very satisfied or satisfied were categorised as 'Responders'.

In addition, MRI images of the affected shoulder were obtained at the 10-year follow-up. Our focus of interest is at full thickness rotator cuff tears according to the Zlatkin/Neumann classification (ref).

Statistical analysis for the primary study question (ASD vs. Placebo surgery) and the pragmatic comparison (ASD vs. ET) at the 10-year follow-up

The trial is primarily designed to ascertain whether ASD was superior to diagnostic arthroscopy for pain reduction (two primary outcomes) after the procedure (the primary confirmatory comparison). We also included a pragmatic comparison of the relative benefits of ASD vs exercise therapy (the secondary exploratory comparison), with the two primary outcomes. All analyses will be performed according to this statistical analysis plan by an independent statistician.

We will quantify the treatment effect on an intention to treat (ASD vs diagnostic arthroscopy comparison) or full analysis set (ASD vs exercise therapy comparison) basis as the difference between the groups in pain scores (VAS), CM score, simple shoulder test score, 15D score and SF-36 score with the associated 95% CIs and p values at 10 years after the primary randomisation. In intention-to-treat and full analysis set analyses, the participants were included as randomised. We will use a mixed-model repeated

measurements analysis of variance with participant as a random factor (repeated measurements at 3, 6, 12, 24 months, 5 years, and 10 years), the baseline value as a covariate, and assuming a covariance structure with compound symmetry. As the mixed-model repeated measurements analysis of variance allows for analysis of unbalanced data sets without imputation, we analysed all available data, the full analysis set. The missingness of the outcome data at different time points will be shown in tabular format. We will fit the mixed-model repeated measurements model by using the mixed procedure in Stata and used Satterthwaite's method to calculate the degrees of freedom. We will use generalised estimating equation logistic regression analysis to analyse categorical variables. We will compare the frequencies of patients who report satisfaction or subjective improvement, the proportions of responders and nonresponders based on patients' satisfaction with the treatment outcome, and the incidence of treatment group unblindings, treatment conversions, and reoperations between the two groups at 10 years.

To safeguard against potential multiplicity effects in the primary comparison, we will require a statistically significant treatment effect for both primary outcome variables. All secondary analyses will be supportive, exploratory and/or hypothesis-generating. We will carry out two sensitivity analyses (the per protocol and as treated) with the same principles as the intention-to-treat and full analysis set analyses. The perprotocol population is the subset of the intention to treat population who received the treatment they were randomised to and who did not receive any other treatment, that is, the patients with a treatment conversion have been excluded (ASD: n=59, diagnostic arthroscopy: n=54). The as treated population is defined according to the treatment the participants received, that is, the 9 participants who originally received diagnostic arthroscopy and the 17 participants who originally received exercise therapy, but due to persistent symptoms requested unblinding and subsequently received ASD, have been included in the ASD population (ASD: n=86, diagnostic arthroscopy: n=54). We will consider a p value of 0.05 to indicate statistical significance. Stata V.15.1 (StataCorp, USA) will be used for all statistical analyses.

Analysis plan for the secondary study question (ASD vs. no ASD) at the 10-year follow-up

In addition to the primary study question whether ASD is superior to diagnostic arthroscopy for pain reduction, a secondary study question was delineated to assess whether ASD confers protective effects against the occurrence of rotator cuff tears and subsequently patient rated outcomes. Given the noteworthy instances of treatment conversions from both the exercise therapy (n=17) and diagnostic arthroscopy (n=9) groups to undergo ASD, juxtaposed with the occurrences of participant withdrawals and loss to follow-up, we decided to compare the prevalence of full thickness rotator cuff tears and PROMs of the subjects who had underwent ASD at any time point during the 10-year follow-up and of those who did not undergo the ASD surgery. The numbers available for this analysis, i.e., the number of patients with MR images at the 10-year follow up are 68 in the ASD group and 80 in the comparison (non-ASD) group.

To be noted, a clear prognostic imbalance exists between the two interventions owing to the exclusions carried out before the second randomisation in the group primarily allocated to surgery: 17 (12%) of the 139 participants allocated to the two surgical groups were excluded without any comparable exclusions from the exercise therapy group. Thus, the ASD versus non-ASD comparison is likely to be biased in favour of ASD owing to the systematic removal of patients from the ASD and Placebo surgery groups with likely poorer prognosis.

As a full thickness rotator cuff tear (0/1), we will consider consensus rating of full thickness tear in the MRI, based on the Zlatkin/Neumann classification by three experienced readers or secondary surgery for rotator cuff tear at any time during the follow-up. There are 13 patients with full thickness rotator cuff tears at 10 years.

As the PROMs we will assess the same outcomes as regards our primary study question.

Statistical analysis for the secondary study question

We acknowledge that comparing rotator cuff tear rates between patients who received ASD and those who did not will be a secondary exploration and underpowered. Nevertheless, our analysis will focus on comparing the proportion of full thickness rotator cuff tears between the ASD and non-ASD groups with a Chi-squared test.

Prognostic imbalances between the two groups add complexity to the analysis. Specifically, 12% of patients in the ASD and Placebo surgery groups combined were excluded in the operating theatre before randomization, whereas no similar exclusions occurred in the ET group. Nevertheless, we will primarily compare the ASD and non-ASD groups without any adjustments for the prognostic imbalance, but we will acknowledge the prognostic imbalance in the interpretation of the results.

ST
